# Probing the limits of plasmonic enhancement using a two-dimensional atomic crystal probe

**DOI:** 10.1038/s41377-018-0056-3

**Published:** 2018-08-29

**Authors:** Wen Chen, Shunping Zhang, Meng Kang, Weikang Liu, Zhenwei Ou, Yang Li, Yexin Zhang, Zhiqiang Guan, Hongxing Xu

**Affiliations:** 1 School of Physics and Technology, Center for Nanoscience and Nanotechnology, and Key Laboratory of Artificial Micro- and Nano-structures of Ministry of Education, Wuhan University, Wuhan, 430072 China; 2 The Institute for Advanced Studies, Wuhan University, Wuhan, 430072 China

## Abstract

Achieving larger electromagnetic enhancement using a nanogap between neighboring metallic nanostructures has been long pursued for boosting light–matter interactions. However, the quantitative probing of this enhancement is hindered by the lack of a reliable experimental method for measuring the local fields within a subnanometer gap. Here, we use layered MoS_2_ as a two-dimensional atomic crystal probe in nanoparticle-on-mirror nanoantennas to measure the plasmonic enhancement in the gap by quantitative surface-enhanced Raman scattering. Our designs ensure that the probe filled in the gap has a well-defined lattice orientation and thickness, enabling independent extraction of the anisotropic field enhancements. We find that the field enhancement can be safely described by pure classical electromagnetic theory when the gap distance is no <1.24 nm. For a 0.62 nm gap, the probable emergence of quantum mechanical effects renders an average electric field enhancement of 114-fold, 38.4% lower than classical predictions.

## Plasmonics: 2D materials help antennas find their hotspot

Metallic nanostructures that localize and manipulate light can be optimized using tiny flakes of inorganic crystals. In ‘nanogap’ antenna systems, intense plasmonic fields capable of harvesting light are created by placing ultra-small metal objects as close to each other as possible. Shunping Zhang and colleagues at China’s Wuhan University have used spectroscopic techniques to determine the nanomaterial separations that maximize plasmonic fields before effects such as electron tunneling become dominant. The researchers turned to molybdenum disulfide (MoS_2_)—a graphene-like, two-dimensional semiconductor capable of handling the damaging hotspots formed in the gap—to tune the distance between a gold nanoparticle and a smooth gold film. By inserting different numbers of MoS_2_ layers between the metals, the team quantitatively identified ideal nanogaps by measuring the intensity of surface-enhanced Raman scattering signals.

## Introduction

Plasmonic field enhancement serves as one of the most attractive phenomena in nanophotonic systems for boosting light–matter interactions^1,2^. This remarkable feature enables various advanced optical applications, including single-molecule surface-enhanced spectroscopy^3–10^, enhanced nonlinearity^11,12^, optical sensing^13–15^, light–matter strong coupling^16^, and nanolasing^17^. Adjacent metallic nanostructures serve as a plasmonic antenna for efficient light harvesting and concentration, typically with orders of magnitude field enhancement in the gap region^1,2,5,11^. To obtain higher plasmonic enhancement, the gap distance in a plasmonic antenna should be as narrow as possible, as predicted by Maxwell’s equations. However, as the gap distance reaches Ångström scale, further narrowing the gap distance will result in saturation or weakening of the plasmonic enhancement due to the appearance of nonlocal screening or electron tunneling^18–25^. Thus, a nanogap antenna should have the optimum gap distance to reach its maximum plasmonic enhancement. However, the quantitative probing of this quantum-limited plasmonic enhancement remains a challenge task; previous demonstrations of the quantum mechanical effects within a tiny gap have typically relied on indirect measurements of far-field scattering spectra^20,21,23^, which usually provides a good hint but is in principle different from the probing of the near-field enhancement.

Measuring the surface-enhanced Raman scattering (SERS) of probe molecules situated inside the gap area of a nanoantenna provides a convenient way of reporting the plasmonic enhancement, qualitatively^26,27^. The reason is that, generally, the electromagnetic enhancement dominates the contributions of the measured SERS enhancement factor (EF), which approximately follows the fourth power of the local electric field enhancement^28^. To date, quantitatively probing the plasmonic enhancement in a nanogap antenna by SERS still faces several difficulties. First, the gap distances between the nanostructure surfaces should be precisely controlled to the subnanometer length scale in three dimensions, a challenge for nanofabrication and characterization techniques. Second, the size of probe molecules (such as benzene derivatives) are usually similar with or even larger than the gap distance between closely separated nanostructures, which increases the difficulty of inserting a probe into the “hotspot”. As a result, the number of molecules within the gap region, a key parameter in calculating the SERS EF, cannot be precisely determined. Third, the orientation of the probe molecules inside the gap region is difficult to control, preventing the alignment of molecular vibration with respect to the strongest plasmonic field component. If the probe molecules must lie down to squeeze into the gap, the measured SERS EF should be reduced due to the orthogonal orientation of molecular vibration and the local field, regardless of whether quantum mechanical effects are present.

**Fig. 1:**
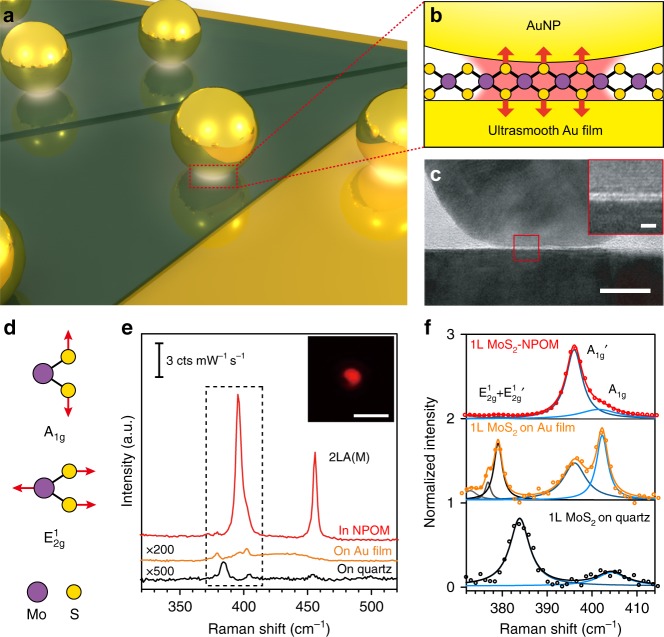
Probing directional plasmonic enhancements using a two-dimensional atomic crystal probe. **a** 3D schematic of different number of layers MoS_2_ spaced NPOMs. **b**, **c** Schematic (**b**) and high-resolution TEM (**c**) images of a 1L MoS_2_-NPOM cross-section in its nanocavity region. The inset in **c** shows the enlarged image of the marked area (red square). Scale bars in **c** and in its inset are 10 nm and 1 nm. **d** Atomic displacements of the A_1g_ and ${\mathrm{E}}_{2{\mathrm{g}}}^1$ modes in the unit cell of 1 L MoS_2_. **e** Raman scattering spectra of a 32-nm-thick Al_2_O_3_ coated 1 L MoS_2_-NPOM, a 1 L MoS_2_ on ultrasmooth gold film and on quartz, respectively. The inset shows the Raman imaging at 396 ± 10 cm^−1^, scale bar, 2 μm. **f** Normalized spectra taken from the black dot region in **d**, including the Lorentz fitting

Here, we develop a MoS_2_ spaced nanoparticle-on-mirror (labeled as MoS_2_-NPOM) plasmonic antenna system to overcome these drawbacks and to probe the limits of the plasmonic field enhancement by quantitative SERS. Single- and few-layer MoS_2_ are used as a spacer, as well as a two-dimensional atomic crystal probe situated between a gold nanoparticle (AuNP) and an ultrasmooth gold film. The gap distance of the MoS_2_-NPOM is precisely tuned by the number of layers of the MoS_2_ interlayer in intervals of 0.62 nm (Fig. 1a). As a SERS probe, the MoS_2_ interlayer is filled into the gap area with a fixed lattice orientation over the entire gap area. Incident light is effectively confined into this nanocavity, exciting a highly localized plasmonic mode with strong electric field intensity, which greatly enhances the lattice vibrations of the analyte MoS_2_ (Fig. 1b). Based on these unique designs, we realize the quantitative probing of the vertical and horizontal field enhancements in a subnanometer gap antenna system by measuring the SERS enhancement of the out-of-plane and the in-plane lattice vibrations of the MoS_2_, respectively. By redshifting the plasmon resonance peak to the excitation laser wavelength, the maximum surface-averaged SERS EF for each MoS_2_-NPOM can be obtained quantitatively. This maximum SERS EF increases with the decrease in the layer number of MoS_2_ and reaches a maximum value of 1.7 × 10^8^ for the out-of-plane phonon modes, corresponding to a 114-fold enhancement in the vertical local field. Theoretical calculations based on pure classical Maxwell’s descriptions can predict the measured plasmonic enhancement quite well when the gap separation is no <1.24 nm, but they overestimate the field enhancement at shorter gap distances. These features of the narrow gap match well with the prediction of quantum-corrected model^18,19,22^, suggesting the probable emergence of electron tunneling across the monolayer (1L) MoS_2_. Our studies could enable further quantitative study of directional field enhancements in plasmonic systems using SERS, and could guide various applications, including surface-enhanced spectroscopy, plasmon-enhanced photon–phonon interaction^29–31^, and Raman-based optomechanics^32,33^.

## Results

### Single atomic layers as two-dimensional crystal probes

To quantitatively probe plasmonic enhancement using SERS, structural parameters that strongly affect SERS EF calculations should be defined as clearly as possible^26,27^. First, the geometry of plasmonic antennas should be precisely characterized, particularly the exact shape of the nanogaps. The gap region of a typical 1L MoS_2_-NPOM is displayed in transmission electron microscopy (TEM) cross-sectional images (Fig. 1c). The structure consists of an ultrasmooth gold film with 0.32 nm root-mean-square roughness (Supplementary Fig. S1), a 1 L MoS_2_ with a precisely defined thickness of 0.62 nm and a 50 nm AuNP (see Materials and Methods for sample fabrication). The atomic migration effect induced by the interaction between the Au and the MoS_2_ flattens the bottom region of the AuNP^34^, leading to an average facet size of 19.4 nm (Supplementary Fig. S2). The citrate molecules loosely covering the AuNP surface are nearly invisible to TEM, as the measured average gap distance of the nanocavity equals the thickness of the 1L MoS_2_ (see detailed discussions in Supplementary Fig. S2). Therefore, the MoS_2_-NPOM system provides a robust nanocavity that allows for the quantitative determination of the area of the “hotspot” and the gap distance.

Second, the SERS probes should lie exactly in the “hotspot”, with known orientations with respect to the nanogap axis. The NPOM geometry^20,30,34–36^ combined with a two-dimensional atomic crystal probe of MoS_2_ can perfectly address these issues. On one hand, the MoS_2_ probe uniformly fills the entire gap of the nanocavity, where the local field is maximized. The exact area of the MoS_2_ probe (corresponding to the number of conventional probe molecules) inside the “hotspot” is naturally determined by the size of the nanocavity. On the other hand, the single atomic layer of MoS_2_ has a definite lattice orientation such that its out-of-plane (A_1g_) and in-plane (${\mathrm{E}}_{2{\mathrm{g}}}^1$) lattice vibrations are aligned with the vertical and horizontal local fields of the MoS_2_-NPOM, respectively (Fig. 1b-d). The MoS_2_-NPOM enhanced Raman scattering intensity *I*_SERS_ can expressed as $I_{{\mathrm{SERS}}} \propto | {\mathop {\alpha }\limits^ \leftrightarrow (\omega _{\mathrm{R}},\omega ){\mathbf{E}}(\omega )} |^2$, where **E**(*ω*) is the local electric field induced by the incident field, and $\mathop {\alpha }\limits^ \leftrightarrow (\omega _{\mathrm{R}},\omega )$ is the Raman polarizability tensor. For 1L MoS_2_, the expression reads as follows:

$$\mathord{\buildrel{\lower3pt\hbox{$\scriptscriptstyle \leftrightarrow$}} \\ \over \alpha } ^{\mathrm{E}}\left( {\omega _{\mathrm{R}},\omega } \right) 	= \left( {\begin{array}{*{20}{c}} {\alpha _{xx}^{\mathrm{E}}} & {\alpha _{xy}^{\mathrm{E}}} & 0 \\ {\alpha _{yx}^{\mathrm{E}}} & {\alpha _{yy}^{\mathrm{E}}} & 0 \\ 0 & 0 & 0 \end{array}} \right)\,{\mathrm{and}}\,\mathord{\buildrel{\lower3pt\hbox{$\scriptscriptstyle \leftrightarrow$}} \\ \over \alpha } ^{\mathrm{A}}\left( {\omega _{\mathrm{R}},\omega } \right) \\ 	= \left( {\begin{array}{*{20}{c}} {\alpha _{xx}^{\mathrm{A}}} & 0 & 0 \\ 0 & {\alpha _{yy}^{\mathrm{A}}} & 0 \\ 0 & 0 & {\alpha _{zz}^{\mathrm{A}}} \end{array}} \right)$$

with $\alpha _{xx}^{\mathrm{E}} = \alpha _{xy}^{\mathrm{E}} = \alpha _{yx}^{\mathrm{E}} = - \alpha _{yy}^{\mathrm{E}}$ for the ${\mathrm{E}}_{2{\mathrm{g}}}^1$ mode and $\alpha _{xx}^{\mathrm{A}} = \alpha _{yy}^{\mathrm{A}}$ for the A_1g_ mode^37^. The equations immediately imply that the Raman intensity of the in-plane phonon ${\mathrm{E}}_{2{\mathrm{g}}}^1$ is solely determined by the horizontal local fields (*E*_*x*_ and *E*_*y*_), whereas the out-of-plane phonon A_1g_ is contributed mainly by *E*_*z*_ because the vertical local field is dominant in the MoS_2_-NPOM. Based on our further analysis (see Supplementary Note S1), the horizontal (vertical) plasmonic enhancement of the MoS_2_-NPOM can be quantitatively obtained by the SERS EF from the ${\mathrm{E}}_{2{\mathrm{g}}}^1$ (A_1g_) phonon. Figure 1e shows the Raman spectra obtained from a 1L MoS_2_ on quartz, a 1L MoS_2_ on ultrasmooth gold film and a 1L MoS_2_-NPOM with a 32-nm-thick Al_2_O_3_ surface coating. An enlarged view of the spectral region around the distinct phonons is shown in Fig. 1f to better compare the peak positions and shapes. When few-layer MoS_2_ contacts well with the gold film, a doping effect and a local mechanical strain effect will occur^38,39^. As a result, both the ${\mathrm{E}}_{2{\mathrm{g}}}^1$ and A_1g_ are softened, with two new peaks appearing near the red side of the ${\mathrm{E}}_{2{\mathrm{g}}}^1$ and the A_1g_, labeled the ${\mathrm{E}}_{2{\mathrm{g}}}^1$′ and A_1g_′ modes (Fig. 1f and Supplementary Fig. S3a). Based on handedness-resolved Raman measurements (Supplementary Fig. S3), the ${\mathrm{E}}_{2{\mathrm{g}}}^1$′ (A_1g_′) is considered split from the ${\mathrm{E}}_{2{\mathrm{g}}}^1$ (A_1g_). Therefore, we sum the ${\mathrm{E}}_{2{\mathrm{g}}}^1$′ (A_1g_′) and ${\mathrm{E}}_{2{\mathrm{g}}}^1$ (A_1g_) and label them ${\mathrm{E}}_{2{\mathrm{g}}}^1$+${\mathrm{E}}_{2{\mathrm{g}}}^1$′ (A_1g_+A_1g_′) modes in the following analysis. Comparing the Raman spectra of the 1L MoS_2_-NPOM with that of the 1L MoS_2_ on an ultrasmooth gold film, we find that both the A_1g_+A_1g_′ and ${\mathrm{E}}_{2{\mathrm{g}}}^1$+${\mathrm{E}}_{2{\mathrm{g}}}^1$′ are largely enhanced in the MoS_2_-NPOM, whereas the spectral intensity of the former is approximately two orders of magnitude higher than that of the latter (Fig. 1e). These results guarantee the validity of using the ${\mathrm{E}}_{2{\mathrm{g}}}^1$+${\mathrm{E}}_{2{\mathrm{g}}}^1$′ and A_1g_+A_1g_′ phonons in probing the horizontal and vertical field enhancements in the MoS_2_-NPOM nanocavity (Supplementary Note S1). Raman imaging of the A_1g_+A_1g_′ (396 ± 10 cm^−1^) shows a bright spot at the position of the AuNP, confirming that the enhancement originates from the MoS_2_-NPOM nanocavity (the inset of Fig. 1e).

### Far-field and near-field analysis of MoS_2_-NPOM antenna

**Fig. 2:**
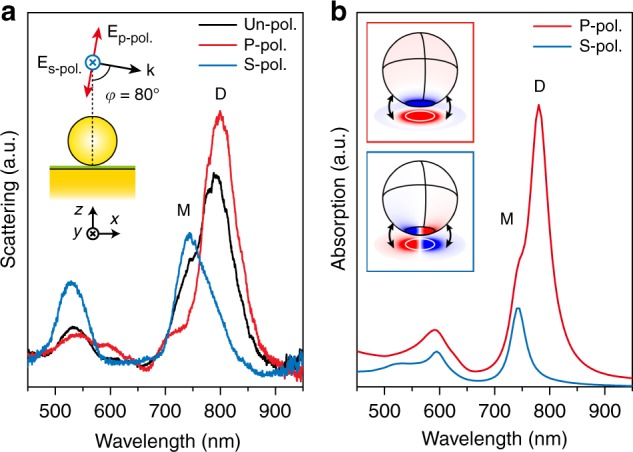
Plasmonic modes analysis. **a**, **b** Measured dark-field scattering (**a**) and simulated absorption (**b**) spectra of a 1 L MoS_2_-NPOM with 32-nm-thick Al_2_O_3_ surface coating excited by oblique incident white light with different polarizations. The inset in **a** shows the excitation configuration both applied in experiments and simulations. The inset in **b** with red (blue) square shows the surface charge distribution at the D (M) peak excited by p- (s-) polarized light, where the nanocavity is plotted with opened geometry in order to see the charges clearly (black arrows)

To optimize the SERS efficiency, the polarization of incident light should be aligned with the nanogap axis. In the NPOM geometry^20,30,34–36^, the nanoparticle interacts with its electromagnetic image induced under the metallic mirror, in analogy with a nanoparticle dimer antenna with a fixed nanogap axis along the surface normal. In our SERS measurements, the MoS_2_-NPOM is excited by a slightly focused 785 nm laser beam illuminating from the side with an angle of 80° from the normal to the sample (see Materials and methods and Supplementary Fig. S4a). Here, the incident light with polarization parallel to the incident plane, labeled p-polarized light, is expected to excite the nanogap plasmons efficiently. To clarify the contributions from the plasmonic gap modes excited in the SERS measurements, we performed dark-field scattering measurements on the MoS_2_-NPOMs using the same configuration, except with the laser replaced with white light (Materials and methods, the inset of Fig. 2a and Supplementary Fig. S4b).

Figure 2a shows the dark-field scattering spectra of the same 1L MoS_2_-NPOM shown in Fig. 1e with un-polarized, p-polarized, and s-polarized incident light. The spectra manifest two peaks at ~785 nm, which are labeled M mode (743 nm) and D mode (798 nm). The M mode can be activated by both p- and s-polarized light, whereas the D mode can only be excited under p-polarized excitation. The abovementioned far-field behaviors of the D and M modes are also confirmed by the corresponding simulated polarization-dependent absorption spectra with a similar excitation configuration (Materials and Methods and Fig. 2b). The insets of Fig. 2b show the surface charge distributions of the D (M) mode excited by p- (s-) polarized light. For the D mode, one charge is localized on the entire bottom facet, and the opposite charge is distributed on the remaining part of the AuNP surface, as well as on the gold film surface beneath the AuNP. This charge pattern, which can only be activated by p-polarized light, suggests that the D mode is a strongly radiating antenna mode^2,40^. In the case of the M mode, whereas the top surface of the AuNP is left nearly blank, both positive and negative charges are mainly concentrated on the two halves of the AuNP’s bottom facet with a blank node at the centre. A similar pattern with opposite charges is induced on the surface of the subjacent gold mirror. The M mode can be understood as the lowest frequency cavity mode^15,40,41^, which appears in parallel flat terminals formed in the MoS_2_-NPOM geometry. This tightly confined charge distribution in the gap region makes the M mode insensitive to the refractive index change on top of the AuNP but ultrasensitive to that inside the nanocavity^15^.

**Fig. 3:**
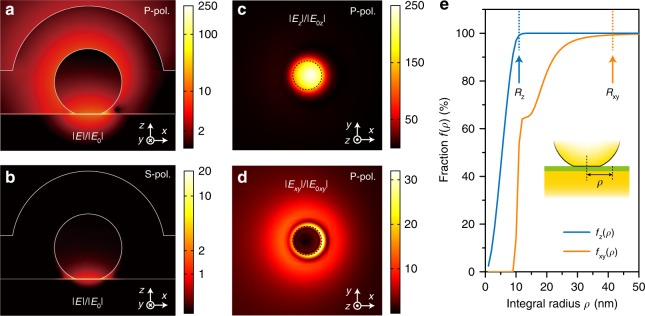
Local field distributions of MoS_2_-NPOM and effective areas of MoS_2_ probe. **a**, **b** Electric field distributions at 785 nm of a 32-nm-thick Al_2_O_3_ coated 1 L MoS_2_-NPOM excited by p-polarized (**a**) and s-polarized (**b**) light. The MoS_2_-NPOM cross-sections are taken from a plane 5 nm away from the xz plane. **c**, **d** Vertical (**c**) and horizontal (**d**) components of the electric field distribution in the 1 L MoS_2_-NPOM with p-polarization excitation. **e** Fraction *f*_*z*_ (*f*_*xy*_) as a function of integral radius *ρ*_*z*_ (*ρ*_*xy*_). The inset in **e** shows the definition of integral radius *ρ*. The effective vertical (horizontal) local field area ${\mathrm{\pi }}R_z^2$ (${\mathrm{\pi }}R_{xy}^2$) is determined by setting *ρ*_z_= *R*_*z*_ (*ρ*_xy_=*R*_*xy*_) for *f*_z_ (*R*_*z*_) = *f*_xy_ (*R*_*xy*_) = 1-e^−5^ (~99.3%)

The electric field distributions of the 1L MoS_2_-NPOM at 785 nm excited by p-polarized and s-polarized light are shown in Fig. 3a, b. For p-polarization excitation, both the D and M modes are excited, but the latter shows greater detuning with respect to the laser wavelength. The modes provide intense electric field enhancement, with a maximum enhancement of 240-fold, where the contribution of the M mode is negligible in the SERS process. For s-polarization excitation, the D mode cannot be excited, while the M mode can still be effectively excited, providing a 20-fold maximum electric field enhancement. The results agree well with the measured polarization-dependent SERS from the MoS_2_-NPOMs shown in Supplementary Fig. S4d. We determined the average vertical (horizontal) plasmonic enhancement $\bar g_z( {\bar g_{xy}} )$ based on the approximation that the SERS EF is proportional to the fourth order of the local electric field enhancement in the MoS_2_-NPOM^28^. Specifically, The enhancement is associated with the surface-averaged vertical or horizontal SERS EF as follows (see details in Supplementary Note S1):

1 $$\overline {{\mathrm{EF}}} _z = \frac{{I_{{\mathrm{SERS}}}^{\mathrm{A}}{\mathrm{/}}S_{{\mathrm{SERS}}}^z}}{{I_{{\mathrm{Ref}}}^{\mathrm{A}}{\mathrm{/}}S_{{\mathrm{Ref}}}}} \approx \left| {\bar g_z} \right|^4$$

2 $$\overline {{\mathrm{EF}}} _{xy} = \frac{{I_{{\mathrm{SERS}}}^{\mathrm{E}}{\mathrm{/}}S_{{\mathrm{SERS}}}^{{\mathrm{xy}}}}}{{I_{{\mathrm{Ref}}}^{\mathrm{E}}{\mathrm{/}}S_{{\mathrm{Ref}}}}} \approx \left| {\bar g_{xy}} \right|^4$$

where $I_{{\mathrm{SERS}}}^{\mathrm{A}}$ ($I_{{\mathrm{SERS}}}^{\mathrm{E}}$) and $S_{{\mathrm{SERS}}}^z$ ($S_{{\mathrm{SERS}}}^{xy}$) are the Raman scattering intensity and the effective “hotspot” area of the A_1g_+A_1g_′ (${\mathrm{E}}_{2{\mathrm{g}}}^1$+${\mathrm{E}}_{2{\mathrm{g}}}^1$′) in the MoS_2_-NPOM, and $I_{{\mathrm{Ref}}}^{\mathrm{A}}$ ($I_{{\mathrm{Ref}}}^{\mathrm{E}}$) and *S*_Ref_ are the Raman scattering intensity and the excitation area of the A_1g_ (${\mathrm{E}}_{2{\mathrm{g}}}^1$) for the same layer MoS_2_ on quartz. *S*_Ref_ is established as the collection area (~3.2 μm^2^) because the excitation beam is larger than the collection area in our experiments. Because the “hotspot” dominates the SERS signal, the effective local field areas $S_{{\mathrm{SERS}}}^z$ and $S_{{\mathrm{SERS}}}^{xy}$ should be much smaller than the collection area and can be determined based on the local field distribution of the MoS_2_-NPOM. Figure 3c, d shows the vertical and horizontal electric field distributions of a MoS_2_-NPOM with structural parameters corresponding to those in Fig. 1. The results suggest that the vertical local field is mostly localized in the area below the AuNP’s circular bottom facet and decays rapidly outside the nanocavity region (Fig. 3c). In contrast, the horizontal local field is distributed almost entirely outside the facet region, with only 9.7% SERS EF contributing from the area directly below the bottom facet of the AuNP and the rest from the outer region with a longer decay length (Fig. 3d). Here, the $S_{{\mathrm{SERS}}}^z$ ($S_{{\mathrm{SERS}}}^{xy}$) is treated as a circular area with radius *R*_*z*_ (*R*_*xy*_), which can be calculated by defining a fraction *f*_*z*_ (*f*_*xy*_) as the ratio of the vertical (horizontal) SERS EF contributed from a circular area ${\mathrm{\pi }}\rho _z^2$ (${\mathrm{\pi }}\rho _{xy}^2$) centered at the nanogap region (the inset of Fig. 3e) to that from the collection area (considered as infinity):

3 $$f_z \left(\rho _z\right) = {\int}_{\!\!\!\! 0}^{{\mathrm{\pi }}\rho _z^2} {\left| {E_z} \right|^4/\left| {E_{0z}} \right|^4{\mathrm{d}}s/{{\int}_{\!\!\!\! 0}^\infty}\left| {E_z} \right|^4/{\left| {E_{0z}} \right|^4{\mathrm{d}}s} }$$

4 $$f_{xy}{\mathrm{(}}\rho _{xy}{\mathrm{) = }}{\int}_{\!\!\!\! 0}^{{\mathrm{\pi }}\rho _{xy}^2} {\left| {E_{xy}} \right|^4/\left| {E_{0xy}} \right|^4{\mathrm{d}}s} {\mathrm{/}}{\int}_{\!\!\!\! 0}^\infty {\left| {E_{xy}} \right|^4/\left| {E_{0xy}} \right|^4{\mathrm{d}}s}\hskip-14pt$$

where *E*_*z*_ (*E*_*xy*_) and *E*_0*z*_ (*E*_0*xy*_) are the vertical (horizontal) electric components of the local and incident field, respectively. *f*_*z*_ (*f*_*xy*_) is depicted as a function of *ρ*_*z*_ (*ρ*_*xy*_) in Fig. 3e, demonstrating that *f*_*z*_ (*f*_*xy*_) saturates quickly with increasing *ρ*_*z*_ (*ρ*_*xy*_). $S_{{\mathrm{SERS}}}^z$ can be obtained by setting *ρ*_*z*_ *=* *R*_*z*_ for *f*_*z*_(*R*_*z*_) = 1−e^−5^ (~99.3%, see Fig. 3e), meaning that this probe area contributes the total SERS signal. In this case, the effective radius of the vertical local field area *R*_*z*_ is 11 nm, only slightly larger than the bottom facet radius of the AuNP. A similar procedure can be applied to the horizontal direction, which yields an effective radius *R*_*xy*_ of 42 nm. After inserting these values into equations (1) and (2), $\overline {{\mathrm{EF}}} _z$ ($\bar g_z$) and $\overline {{\mathrm{EF}}} _{xy}$ ($\bar g_{xy}$) can be determined in a straightforward manner.

### Probing the limits of plasmonic enhancement by plasmon-scanned SERS

To obtain the maximum SERS signal, the plasmonic resonance wavelength of the nanoantenna should overlap with the incident wavelength as well as the outgoing Raman wavelength. In experiments, satisfying this condition is nontrivial, e.g., by wavelength-scanned SERS spectroscopy^7,25,42^. However, this technique requires expensive narrow-line lasers with multiple/tunable wavelengths and careful calibration schemes, preventing its widespread application. Here, we performed a plasmon-scanned SERS measurement to achieve a similar goal: plasmon resonance is gradually redshifted by successively depositing Al_2_O_3_ layers onto the sample surface to match the excitation laser (and the outgoing Raman light) at a fixed wavelength^43^. The effect of the Al_2_O_3_ coating on the near-field distributions of the MoS_2_-NPOM system are shown in Supplementary Fig. S5.

**Fig. 4:**
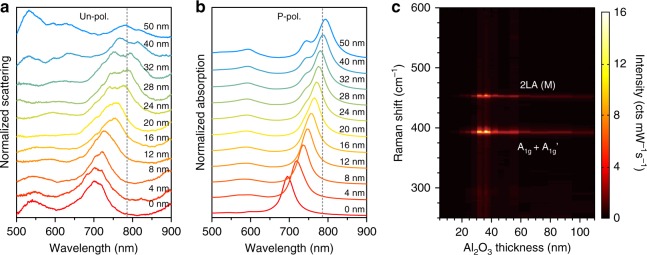
Plasmon-scanned SERS measurement. **a**, **b** Measured dark-field scattering (**a**) and simulated absorption (**b**) spectra of a 1 L MoS_2_-NPOM with the Al_2_O_3_ coating thickness varied from 0 nm to 50 nm. The gray dotted lines in **a** and **b** show the laser line at 785 nm. **c** Intensity map of the SERS spectra of the 1 L MoS_2_-NPOM as the Al_2_O_3_ coating thickness increases from 8 nm to 102 nm

Dark-field scattering spectra of a 1 L MoS_2_-NPOM with different Al_2_O_3_ coating thicknesses are shown in Fig. 4a. The D and M modes are overlapping at ~700 nm without the Al_2_O_3_ coating. The modes show continuous redshift and broadening as the thickness of the Al_2_O_3_ layer increases, resulting from the dielectric screening effect induced by the high refractive index layer. As the Al_2_O_3_ thickness exceeds 20 nm, the D and M modes start splitting into two peaks. The separation originates from the larger spectral shift of the D mode relative to that of the M mode in response to the equal-thickness dielectric coating. This feature can be understood as the result of the distribution of a higher proportion of surface charges on the outer AuNP surface for the D mode than for the M mode (the inset of Fig. 2b). This coating dependence of the resonance wavelength, together with the polarization-dependent dark-field spectra shown in Fig. 2, guarantees an unambiguous identification of the plasmonic resonances in experiments. For comparison, we calculated the corresponding far-field absorption spectra of the MoS_2_-NPOM with p-polarization excitation (Fig. 4b, see the corresponding scattering spectra in Supplementary Fig. S6). The results show similar redshift and broadening behaviors of the D peak, followed by the appearance of the M peak on the blue side of the D peak. The measured and simulated scattering spectra with an Al_2_O_3_ coating exceeds 50 nm; the results of further analysis are shown in Supplementary Fig. S6.

**Fig. 5:**
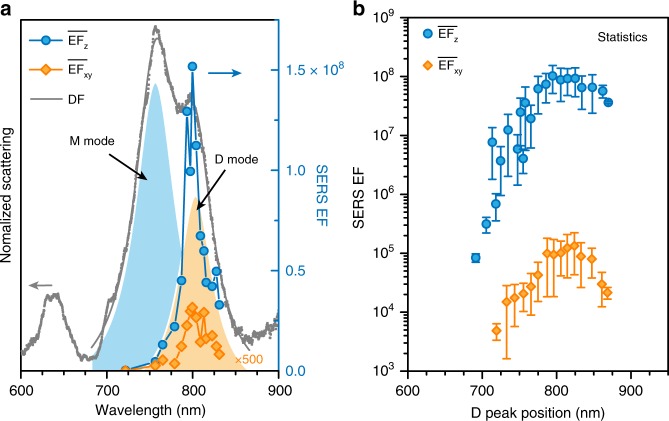
Vertical and horizontal SERS EFs in response to the D peak position. **a** $\overline {{\mathrm{EF}}} _z$ and $\overline {{\mathrm{EF}}} _{xy}$ of a 1 L MoS_2_-NPOM in response to the resonance wavelength of its D mode, along with its dark-field (DF) scattering spectrum at 36-nm**-**thick Al_2_O_3_ coating (where the $\overline {{\mathrm{EF}}} _z$ reaches maximum). The light orange (blue) shadow area represents the far-field shape of the D mode (M mode) by fitting the DF scattering spectrum with Lorentz curves. **b** Statistical average of the $\overline {{\mathrm{EF}}} _z$ and $\overline {{\mathrm{EF}}} _{xy}$ as a function of the D peak position, obtained from 17 individual 1 L MoS_2_-NPOMs. The errors bars represent the standard deviations of the $\overline {{\mathrm{EF}}} _z$ and $\overline {{\mathrm{EF}}} _{xy}$ calculated by averaging the data points of the D peak dependent $\overline {{\mathrm{EF}}} _z$ and $\overline {{\mathrm{EF}}} _{xy}$ with 5 nm wavelength a step

SERS measurements were performed after each Al_2_O_3_ coating under p-polarization excitation below 4 μW/μm^2^ to avoid damage to the sample or possible nonlinear effects. SERS spectra from the same 1L MoS_2_-NPOM are mapped in Fig. 4c, showing a pronounced enhancement of the spectral intensity when the wavelength of the plasmon resonance overlaps that of the excitation laser. For each Al_2_O_3_ coating, we can determine the corresponding peak position of the D mode, $\overline {{\mathrm{EF}}} _z$ and $\overline {{\mathrm{EF}}} _{xy}$ via the dark-field scattering spectrum, equations (1) and (2), respectively. Then, $\overline {{\mathrm{EF}}} _z$ and $\overline {{\mathrm{EF}}} _{xy}$ can be plotted as a function of the resonance wavelength of the D mode, as shown in Fig. 5a. $\overline {{\mathrm{EF}}} _z$ increases rapidly as the D peak wavelength approaches the excitation wavelength and then reaches the maximum value of 1.5 × 10^8^ when the D peak wavelength is between the incoming laser wavelength (785 nm) and the outgoing Raman wavelength (~810 nm). $\overline {{\mathrm{EF}}} _z$ drops quickly when the D peak is red-detuned to the Raman wavelength. This D-peak-scanned $\overline {{\mathrm{EF}}} _z$ profile almost follows the far-field shape of the D mode (Fig. 5a). The corresponding results in the horizontal direction show similar behavior, but the maximum $\overline {{\mathrm{EF}}} _{xy}$ is 6.3 × 10^4^, which is ~2400-fold weaker than that of $\overline {{\mathrm{EF}}} _z$. Similar performances are also observed in the statistical values of $\overline {{\mathrm{EF}}} _z$ and $\overline {{\mathrm{EF}}} _{xy}$ in response to the D peaks (Fig. 5b) obtained from 17 different 1L MoS_2_-NPOMs (Supplementary Fig. S7). As the gap distance in our MoS_2_-NPOM system is precisely determined by the thickness of the MoS_2_, the error bars of $\overline {{\mathrm{EF}}} _z$ mainly originate from the derivation of the diameter and the bottom facet size from different measured AuNPs (Supplementary Fig. S2 and Supplementary Fig. S8). It should be noted that the maximum SERS EFs of several 1L MoS_2_-NPOMs are not included in Fig. 5b because their D peaks become invisible in the dark-field spectra as the Al_2_O_3_ coating thickness exceeds ~70 nm (see details in Supplementary Fig. S6).

Classical electromagnetic simulations predict a smooth curve for the Al_2_O_3_ thickness-dependent $\overline {{\mathrm{EF}}} _z$ (Supplementary Fig. S9), whereas curves in Fig. 5a contain one or several dips near the peak region. A similar feature is observed in other MoS_2_-NPOMs (Supplementary Fig. S7) and the previous wavelength-scanned SERS measurement results^7,25,42^. In conventional single-molecule SERS experiments, large-intensity fluctuation is a typical observation, due to the possible random movements or photodamage of the probe molecules around the ‘hotspot’. In contrast, benefiting from the higher photodamage threshold of the two-dimensional atomic crystal and its strict lattice arrangement in the ‘hotspot’, the SERS spectra of our MoS_2_-NPOM system exhibit high stability and repeatability over time, demonstrating 4.3% intensity fluctuation over 150 minutes (Supplementary Fig. S10a). Therefore, we could exclude the possibility of large-intensity fluctuation during the SERS measurements. Power-dependent SERS measurements show a linear relationship between the incident power and the SERS intensity (Supplementary Fig. S10b), indicating that the process is unlikely to be a stimulated Raman scattering one. Another possible explanation is associated with the phonon nature of the Raman scattering, which may also involve an optomechanical mechanism^32,33,44^. Further studies are required to clarify this feature, which is beyond the scope of the current work.

**Fig. 6:**
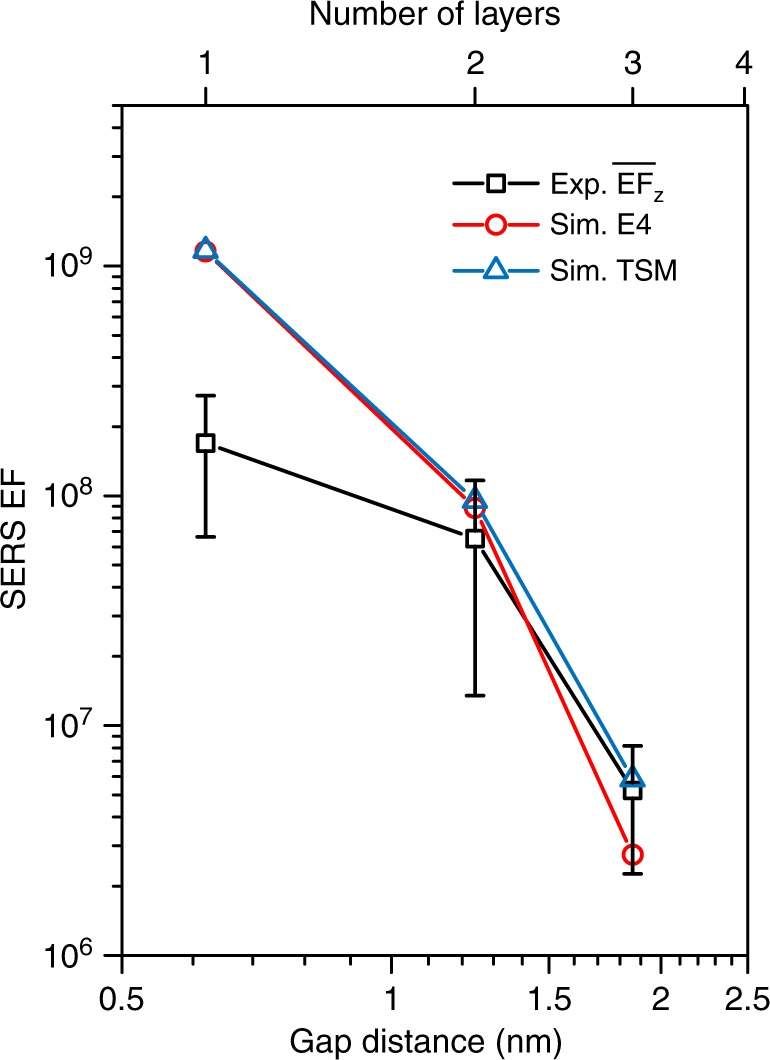
Measured and simulated maximum $\overline {{\mathrm{EF}}} _{\mathrm{z}}$ in response to the gap distance. The error bars represent the standard deviations of the measured maximum $\overline {{\mathrm{EF}}} _{\mathrm{z}}$ averaged from seventeen 1 L, six 2 L and four 3 L MoS_2_-NPOMs. The simulated SERS EFs are calculated by the E4 model and two-study model (TSM), respectively (see Materials and Methods, Supplementary Note S2). The simulated SERS EFs based on the pure classical electromagnetic theories match well with the measured SERS EFs as the gap distance is no less than 1.24 nm, while significantly overestimate the field enhancement at shorter gap distance

Plasmon-scanned SERS measurement can obtain the maximum SERS enhancement of an individual MoS_2_-NPOM, thus enabling quantitative probing of the limits of plasmonic enhancement in nanogaps. We repeated these measurements on six individual bilayer (2 L) MoS_2_-NPOMs and four individual trilayer (3 L) MoS_2_-NPOMs to obtain their maximum SERS EFs. The largest $\overline {{\mathrm{EF}}} _z$ values for 1 L, 2 L, and 3 L MoS_2_ probes reach up to 5.1 × 10^8^, 1.7 × 10^8^, and 8.5 × 10^6^, respectively (Supplementary Fig. S7). The statistical average of the maximum $\overline {{\mathrm{EF}}} _z$ as a function of the gap distance is plotted in Fig. 6, showing that $\overline {{\mathrm{EF}}} _z$ rapidly increases as the gap distance narrows. For comparison, we first calculated the total SERS EFs using the E4 model (see Materials and method, and Supplementary Fig. S9), which can approximate the $\overline {{\mathrm{EF}}} _z$ due to the dominated field enhancement in the vertical direction. The measured and simulated $\overline {{\mathrm{EF}}} _z$ are in good agreement at gap distances of 1.24 nm and 1.86 nm. Thus, our measured $\overline {{\mathrm{EF}}} _z$ can be well described by the electromagnetic enhancement without introducing the concept of chemical enhancement applied in most SERS experiments^27^ and thereby demonstrating the reliability of our designs in probing the plasmonic enhancement. However, this precise prediction starts to overestimate the measured plasmonic enhancement at narrower gap distances. As the gap distance decreases to 0.62 nm, the measured $\bar g_z$ ≈ 114 ($\overline {{\mathrm{EF}}} _z$ = 1.7 × 10^8^) becomes ~38.4% (~6.9-fold) lower than the $\bar g_z$ ≈ 185 ($\overline {{\mathrm{EF}}} _z$ = 1.17 × 10^9^) predicted by the E4 model. Because the gap distance and the orientation of the probe in our antenna system are well-defined and robust, the reduction of the measured plasmonic enhancement in the subnanometer gap is most likely caused by the emergence of previously predicted quantum mechanical effects^18,19,22^. Note that these values are the average values over the total “hotspot” area. The maximum out-of-plane plasmonic enhancement $g_z^{{\mathrm{max}}}$ (at the “hottest” position) can be obtained if the field distribution is precisely known. Taking the geometry variations in the experiments into account, the MoS_2_-NPOM provides a satisfactory linear relationship between the maximum and the surface-averaged SERS EFs: ${\mathrm{EF}}_z^{\mathrm{max}} = 3.24 \times {\mathrm{10}}^8 + 2.5\overline {\mathrm{EF}} _z$ (Supplementary Fig. S8). Therefore, the measured ${\mathrm{EF}}_z^{\mathrm{max}}$ ($g_z^{{\mathrm{max}}}$) is evaluated to be as large as 4.93 × 10^8^ (148).

To further clarify the issue regarding the quenching of the field enhancement, we also introduced a two-study model (TSM) to calculate the SERS EFs (Fig. 6) of the A_1g_ phonon in the MoS_2_-NPOMs and the same layer MoS_2_ on quartz (see Materials and Methods, Supplementary Note S2). Using a polarization current as the source at the Raman frequency, the emission enhancement of the MoS_2_-NPOM can be fully captured^45^. A key difference between molecule vibrations and lattice phonons is the coherence of the Raman signal from different locations, which is characterized by the correlation length. This effect can be qualitatively considered in the TSM by setting the diameter of the MoS_2_ probe equal to the correlation length, as the polarization current is coherent within a MoS_2_ sheet of finite size. Upon assuming the correlation length of the A_1g_ modes in 1 L, 2 L, and 3 L MoS_2_ to be 24 nm, 28 nm, and 34 nm, the SERS EFs obtained by the TSM match those predicted by the E4 model very well (Fig. 6). These assumed correlation lengths are comparable with the measured value of ∼30 nm for optical phonons in graphene^46^. Additionally, the decrease in the correlation length with the decrease in the number of layers is reasonable because the 1 L MoS_2_ may have local wrinkles that disturb the coherence of phonons. Therefore, we can conclude that as the gap distance exceeds 1.24 nm, the two models based purely on classical Maxwell’s descriptions can predict the behaviors of the measured plasmonic enhancement quite well, but they start to overestimate the field enhancement at narrower gap distances. These performances regarding the narrow gap match well with the prediction of the quantum-corrected model, suggesting the probable emergence of electron tunneling across the 1 L MoS_2_ layer.

## Discussion

The horizontal SERS EFs measured by the ${\mathrm{E}}_{2{\mathrm{g}}}^1$ phonon in the MoS_2_-NPOM are larger than the calculated results, which may originate from the gentle ripples in the MoS_2_ layer, as a slight inclination would allow excitation of the in-plane phonons by the intense out-of-plane local electric field in the MoS_2_-NPOM. One may also expect to observe the quenching of the plasmonic enhancement to be reproduced better by using the quantum-corrected model^22,25^. This model is based on the introduction of a gap-distance-dependent tunneling conductivity, which is determined by a phenomenological fit to the data from full quantum mechanical calculations. However, in the MoS_2_-NPOM system, the MoS_2_ is doped by the gold film such that it is nontrivial to obtain the tunneling conductivity. On the other hand, the comparisons between the experiments and the calculations based on the electromagnetic models are sufficiently clear to observe the quantum mechanical effects. Finally, although the MoS_2_ probe can ensure the accuracy of the gap distance and the plasmonic enhancement, the limitation of the MoS_2_-NPOM system is that the gap distance of the antenna can only be controlled stepwise in intervals of the crystal thickness. Graphene or boron nitride is in principle a promising alternative because it comprises a thinner single layer thickness.

In conclusion, we realized the quantitative probing of the vertical and horizontal SERS EFs of MoS_2_-NPOM plasmonic antennas with a gap distance reaching down to a well-defined subnanometer scale. Layered two-dimensional MoS_2_ crystals with strict lattice arrangement were designed as probes inserted into the “hotspot” region to create robust and uniform gaps with intervals of 0.62 nm. Furthermore, directional plasmonic enhancements were extracted by the SERS enhancement of the out-of-plane and in-plane phonons in the MoS_2_ probe. By implementing the plasmon-scanned SERS measurements, we obtained the maximum vertical SERS EF in response to the gap distance that falls within the quantum-limit region. By closely comparing these experiment results with the calculations obtained by two electromagnetic models, we find that the SERS EF of ~10^8^ for gap distances greater than 1.24 nm can be safely described by pure classical electromagnetic theory. For a 0.62 nm gap, the probable emergence of quantum mechanical effects yields a maximum electric field enhancement of 114-fold, 38.4% lower than the classical predictions. Our results provide important insight into advanced applications that rely on optimizing plasmonic enhancement, i.e., single-molecule detections and light–matter strong coupling. We also anticipate that our unique designs could provide an important guide for further understanding quantum mechanical effects as well as plasmon-enhanced photon-phonon interactions and promoting relevant new applications, such as quantum plasmonics and nanogap optomechanics.

## Materials and methods

### Sample fabrication

To develop MoS_2_-NPOM antennas, 1 L, 2 L, and 3 L MoS_2_ layers were exfoliated from bulk MoS_2_ crystals (SPI Supplies) onto an ultrasmooth gold film. The ultrasmooth gold film was fabricated using the template stripping method^47^. Briefly, a 200-nm-thick gold film was evaporated on silicon wafers, then glued to glass slides using optical epoxy (Norland Optical Adhesive 61) via 15 min of UV irradiation. After peeling off the silicon wafer, we obtained a fresh surface of the ultrasmooth gold film. The number of MoS_2_ layers was determined by the contrast of optical images and Raman and photoluminescence spectroscopy. Next, 50 nm citrate-capped AuNPs in aqueous solution (BBI Inc.) were drop-coated onto the area containing the 1 L, 2 L, and 3 L MoS_2_; the sample was then rinsed with deionized water, allowed to sit for 1 min and dried with nitrogen gas. Then, the sample was annealed in a vacuum chamber at 120 °C for 8 h. Finally, Al_2_O_3_ layers were grown on the sample surface with thicknesses varying from 4 nm to 102 nm using atomic layer deposition at 120 °C. The individual MoS_2_-NPOMs were identified by dark-field scattering microscopy and finally verified by scanning electron microscopy (SEM) after all optical measurements (Supplementary Fig. S6).

### SERS spectroscopy

The setup for SERS measurements is shown in Supplementary Fig. S4a. A 785 nm continuous-wave laser was directed through an analyser, a half-wave plate and a cycle aperture, then loosely focused onto the sample by a doublet lens (ƒ = 25 mm) with an angle 80° from the normal to the sample, forming a ∼2500 μm^2^ elliptical beam. The Raman scattering light was collected by a 100 × objective (Olympus, NA = 0.8) and then directed into a Raman spectrometer (Renishaw, inVia) by a flip mirror. For the SERS measurements of the MoS_2_-NPOMs, the collection area was set to ∼1.8 × 1.8 μm^2^, the laser power was ∼9 mW and the integration time was 300 s. Reference Raman spectra were collected from 1 L, 2 L and 3 L MoS_2_ on quartz, and the integration times were 1500 s, 900 s, and 600 s, respectively. The collection area was established as a rectangular area of ∼65 μm^2^, and the laser power was 90 mW. All Raman scattering signals were collected without polarization selection. The peak intensities of the phonon modes were determined by Lorentz fitting.

### Dark-field scattering spectroscopy

The setup for dark-field scattering measurements is shown in Supplementary Fig. S4b. Unpolarized light from a halogen lamp was passed through a spatial filter and then slightly focused on the sample using the same configuration employed in the SERS measurements, forming a broad elliptical beam. The scattered light was collected by the same ×100 objective and directed into the CCD camera (Tucsen, TCH-1.4CICE) for imaging or to the Raman spectrometer (after removing the long-pass filter module) to acquire the dark-field scattering spectra. For polarization-dependent measurements, an analyser was added before the spatial filter to obtain polarization-tunable white light. The collection area was set to ∼1 μm^2^. The integration times of the spectra excited by unpolarized and polarized light were 30 s and 60 s, respectively. The dark-field scattering spectrum was obtained by (S–B)/L, where S is the spectrum of the MoS_2_-NPOM, B is the background spectrum of the nearby same-layer MoS_2_, and L is the spectrum of the incident halogen light with the corresponding polarization.

### Simulations

Full-wave electromagnetic simulations were performed using COMSOL Multiphysics 5.2a, a commercial finite element method package. The MoS_2_-NPOM system was treated as a 50 nm sphere with a bottom facet measuring 19.4 nm in diameter situated on a semi-infinite gold plane separated by 0.62 nm, 1.24 nm, or 1.86 nm thick MoS_2_ layers. First, linearly polarized plane wave excitation with an incident angle of 80° was implemented using a periodic boundary condition in the absence of the nanoparticle. Then, the calculated field was used as the background field for the NPOM configuration, which perfectly matched the layer surrounding the entire simulation domain. The far-field Rayleigh scattering light was collected over a solid angle corresponding to NA = 0.8 to simulate the experimental setup. The permittivity of Au was determined from the experimental data by Johnson and Christy^48^. The refractive indices of quartz and Al_2_O_3_ were both set to 1.5. The in-plane permittivities of 1 L, 2 L and 3 L MoS_2_ were extracted from micro-reflection measurements, whereas the out-of-plane permittivities were set to 1, 2, and 2.4, respectively (see details in Supplementary Fig. S11 and Supplementary Note S3). The SERS EF was first calculated by the E4 model, which was obtained by $\mathop {{\int}{\hskip-2pt}{\int}}\nolimits_{S_{{\mathrm{SERS}}}^z} {\left| {{\mathbf{E}}/{\mathbf{E}}_0} \right|} ^4{\mathrm{d}}s/\mathop {{\int}{\hskip-2pt}{\int}}\nolimits_{S_{{\mathrm{SERS}}}^z} {{\mathrm{d}}s}$. The SERS EF was also simulated by a TSM implemented in COMSOL (see details in Supplementary Note S2). The excitation field was obtained by the same method mentioned above. Then, the emission field at Raman frequency was computed by modeling the MoS_2_ layer as an externally generated polarization **P**, evaluated by the inner product of the Raman polarizability tensor and the local excitation field **E**(**r**). The Raman scattering light was integrated on a spherical surface containing the entire NPOM. Raman scattering from a reference system was evaluated following the same procedures, with a few exceptions: the AuNP and Al_2_O_3_ coating were set to air, the gold film was replaced with a quartz substrate and MoS_2_ disks of finite size were substituted for the MoS_2_ probe.

## Electronic supplementary material

Supplementary Information
