# Supplementary material for: Probing the limits of plasmonic enhancement using a two-dimensional atomic crystal probe
Source: Light Sci Appl. 2018 Aug 29;7:56. doi: 10.1038/s41377-018-0056-3 (PMC6113320; doi:10.1038/s41377-018-0056-3)
Supplement: Supplementary file 1 — Supplementary Information [file 41377_2018_56_MOESM1_ESM.pdf]

Supplementary Information for

Probing the limits of plasmonic enhancement using a two-dimensional atomic crystal probe

Wen Chen[1], Shunping Zhang[1†], Meng Kang[1], Weikang Liu[1], Zhenwei Ou[1], Yang Li[1], Yexin Zhang[1], Zhiqiang Guan[1], Hongxing Xu[1, 2†]

[1]School of Physics and Technology, Center for Nanoscience and Nanotechnology, and Key Laboratory of Artificial Micro- and Nano-structures of Ministry of Education, Wuhan University, Wuhan 430072, China.

[2]The Institute for Advanced Studies, Wuhan University, Wuhan 430072, China.

Email: spzhang@whu.edu.cn; hxxu@whu.edu.cn.

Figure S1. Atomic force microscope characterizations of Au mirrors and MoS_2-NPOMs.
Figure S2. High-resolution TEM characterizations of 1L MoS_2-NPOMs.
Figure S3. Phonon modes analysis of Au doped MoS_2.
Figure S4. Experimental setups and the incident polarization response of A_{1g}+A_{1g}' modes.
Figure S5. Effect of Al_2O_3 coating on the plasmonic response of 1L MoS_2-NPOM
Figure S6. SEM characterizations and dark-field scattering spectra of MoS_2-NPOMs.
Figure S7. Measured $\overline{EF_z}$ of 1L, 2L and 3L MoS_2-NPOMs as a function of Al_2O_3 coating thickness.
Figure S8. Average and maximum SERS EFs in response to the facet size of AuNP.
Figure S9. Simulated SERS EFs of 1L, 2L and 3L MoS_2-NPOMs in response to Al_2O_3 coating thickness calculated by two electromagnetic models.
Figure S10. Stability test and laser power dependence of the SERS intensity of MoS_2-NPOM system.
Figure S11. Dielectric function measurements of single- and few-layer MoS_2 on gold film systems.
Note S1. Analysis of vertical and horizontal SERS EFs.
Note S2. Simulations based on two-study model.
Note S3. Dielectric function measurements and analysis of single- and few-layer MoS_2 on gold film systems.

Supplementary Figures

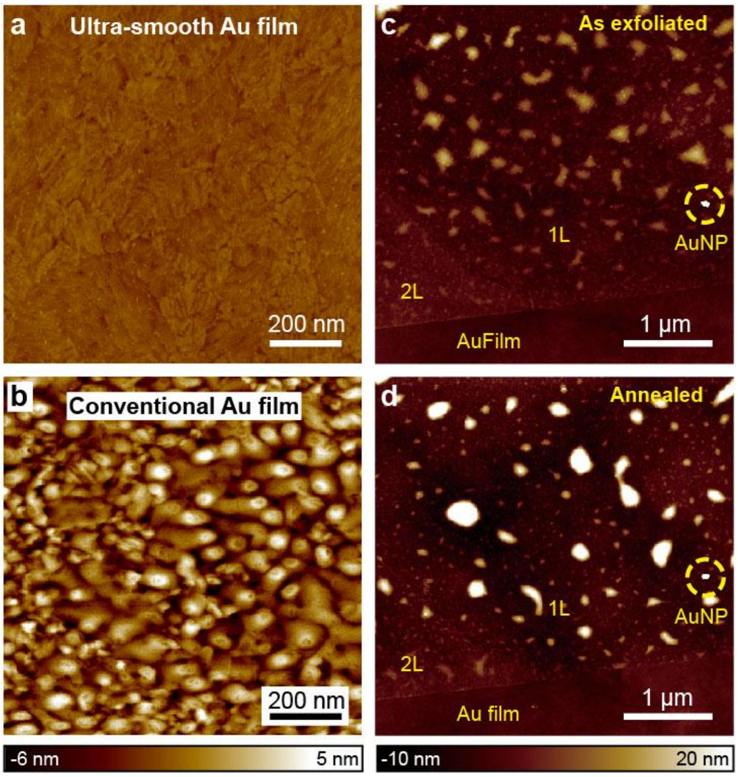

Supplementary Figure S1 Atomic force microscope characterizations of Au mirrors and MoS_2-NPOMs. (**a**, **b**) Atomic force microscope images of ultrasmooth (**a**) and conventional (**b**) gold films. The root-mean-square roughness of the former is 0.32 nm, which significantly smaller than the latter of 1.8 nm. (**c**, **d**) Atomic force microscope images of a sample containing 1L and 2L MoS_2-NPOMs before (**c**) and after (**d**) annealing. For freshly fabricated 1L or few-layer MoS_2 on ultrasmooth gold film, the MoS_2 only loosely contacts with the ultrasmooth gold film. After annealing at 120 ℃ for 8 hours, the MoS_2 turns out to be tightly attached to the gold film, with a part of air remains below the MoS_2 forming bubbles (also shown in Supplementary Figure S2b).

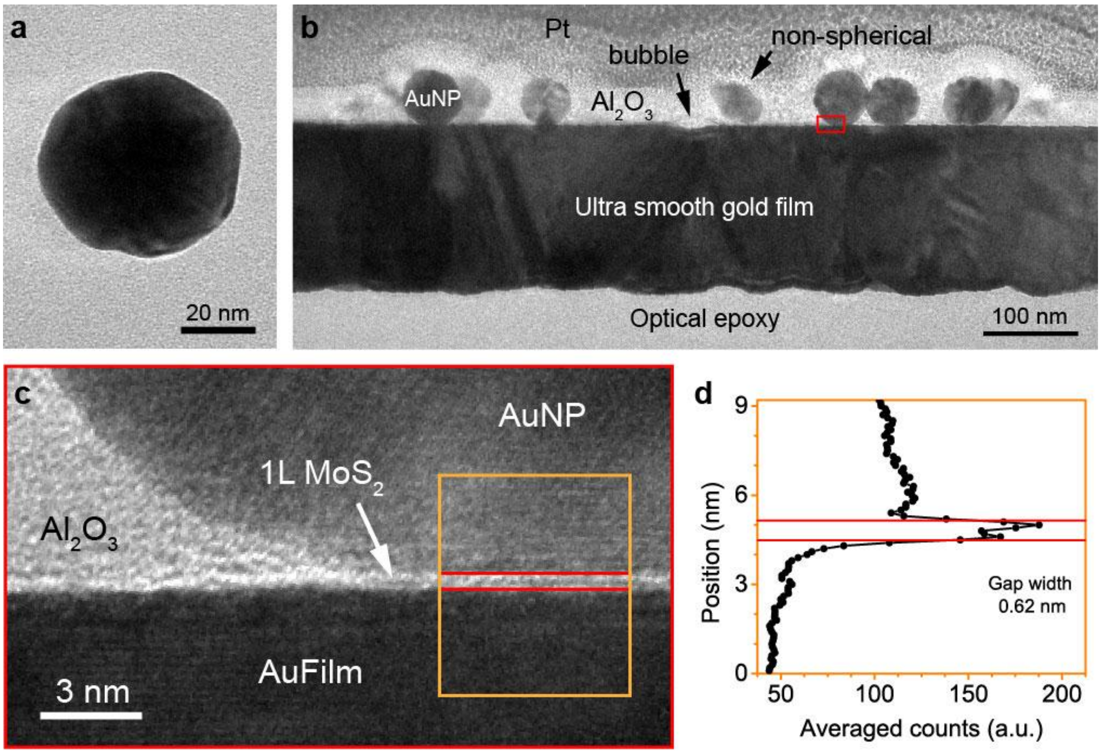

Supplementary Figure S2 High-resolution TEM characterization of 1L MoS_2-NPOMs. (**a**, **b**) TEM images of a typical AuNP (**a**) and a cross-sectional 30-nm-thick Al_2O_3 coated 1L MoS_2-NPOMs (**b**). The average diameter of the AuNP and its bottom facet are 19.4±3.9 nm and 51.2±3.7 nm, which were calculated by measuring 21 spherical AuNPs from 31 observed cross-sectional 1L MoS_2-NPOMs. (**c**) The enlarged images from the marked area in (**b**), showing the gap region of a 1L MoS_2-NPOM. (**d**) Averaged counts profile from the orange selection rectangle in (**c**). As marked by two red lines in (**c**) and (**d**), the measured gap width of the 1L MoS_2-NPOM is 0.62 nm, which agrees well with the theoretical value of 1L MoS_2[1]. The cross-sectional slices containing MoS_2-NPOMs were fabricated as follow procedures. First, a 30-nm-thick Al_2O_3 layer was deposited on the MoS_2-NPOM sample, then the area of interest was protected by a ~300-nm-thick platinum layer using low-voltage electron beam deposition, followed by a several micrometers thick platinum layer coating using ion beam induced chemical vapor deposition (FEI, Versa3D). Next, the area containing individual MoS_2-NPOMs was milled to a 1.5-μm-thick membrane by focused ion beam. After that, the membrane was successively lifted out from the sample and mounted on a TEM grid, and finally thinned to ~100 nm to perform the high-resolution TEM characterization (Titan G2 60-300 Probe Cs Corrector HRSTEM).

There are several factors that seem to bring the extra gap thickness of the MoS_2-NPOMs during the sample fabricating: (1) small molecules from air adsorbed on the surface of the MoS_2 layer, (2) loosely distributed surfactant molecules on the AuNP surface, and (3) the surface roughness of the AuNP and the Au film. However, based on the cross-sectional TEM images, we cannot find the visible molecule spacers or rough areas of the Au surfaces that significantly expand the average gap distance. A possible explanation is that due to the strong interaction between the Au and S atoms, the small molecules were squeezed out from the AuNP/MoS_2 surface during the annealing process. As a result, the bottom shape of the AuNP was changed from the near sphere to the ~19.4-nm-diameter circular plane. In this case, both the Au film and the AuNP tightly contacted with the MoS_2 due to the atom migrations of Au[2], which further reduced the surface roughness of the Au surfaces. Another possible situation is that part of the small molecules and the rough surfaces area were still remained inside the nanocavity regions, which haven't been observed based on the cross-sectional images. In addition, we measured the average gap distance of a cross-sectional 2L MoS_2 on the gold film upon its TEM image. The result shows that the thickness of the 2L MoS_2 is ~1.25 nm, suggesting that the possible expansion effect between the layers is minor in our system.

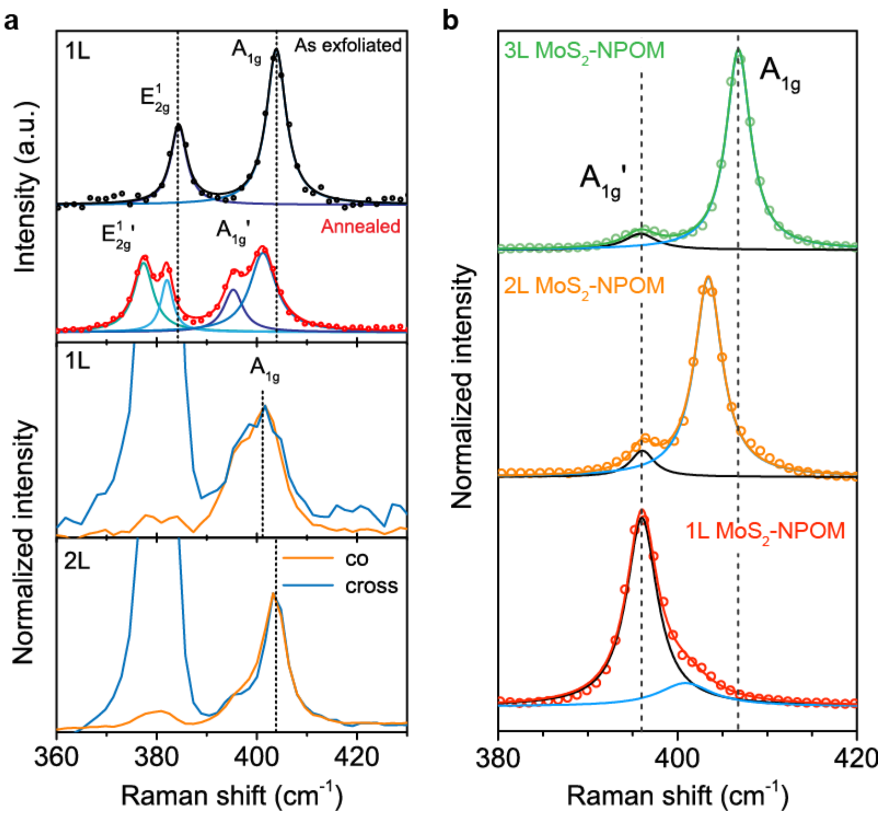

Supplementary Figure S3 Phonon modes analysis of Au doped MoS_2. (**a**) Top panel: Raman scattering spectra of a 1L MoS_2 on ultrasmooth gold film before (black line) and after (red line) annealing, excited by linearly polarized 532 nm laser. Middle and bottom panels: co-circularly (orange line) and cross-circularly (blue line) polarized Raman scattering spectra of a 1L (middle panel) and a 2L (bottom panel) MoS_2 on the gold film, excited by circularly polarized 532 nm light. The handedness-resolved Raman scattering spectra are normalized to the A_{1g}+A_{1g}' intensity. The results show that the A_{1g} and A_{1g}' modes have the same helicity selection rules[3], which supports the assumption that the A_{1g}' mode is split from the A_{1g} mode. (**b**) Normalized Raman scattering spectra of 1L, 2L and 3L MoS_2-NPOMs excited by 785 nm laser. In contrast with the A_{1g} mode, the A_{1g}' mode is independent on the number of layer of MoS_2, with the ratio of A_{1g} to A_{1g}' is significantly decrease as the number of layers increase. These results suggest that the A_{1g}' mode is an out-of-plane mode formed due to the strong interaction between the bottom layer MoS_2 and the Au surface.

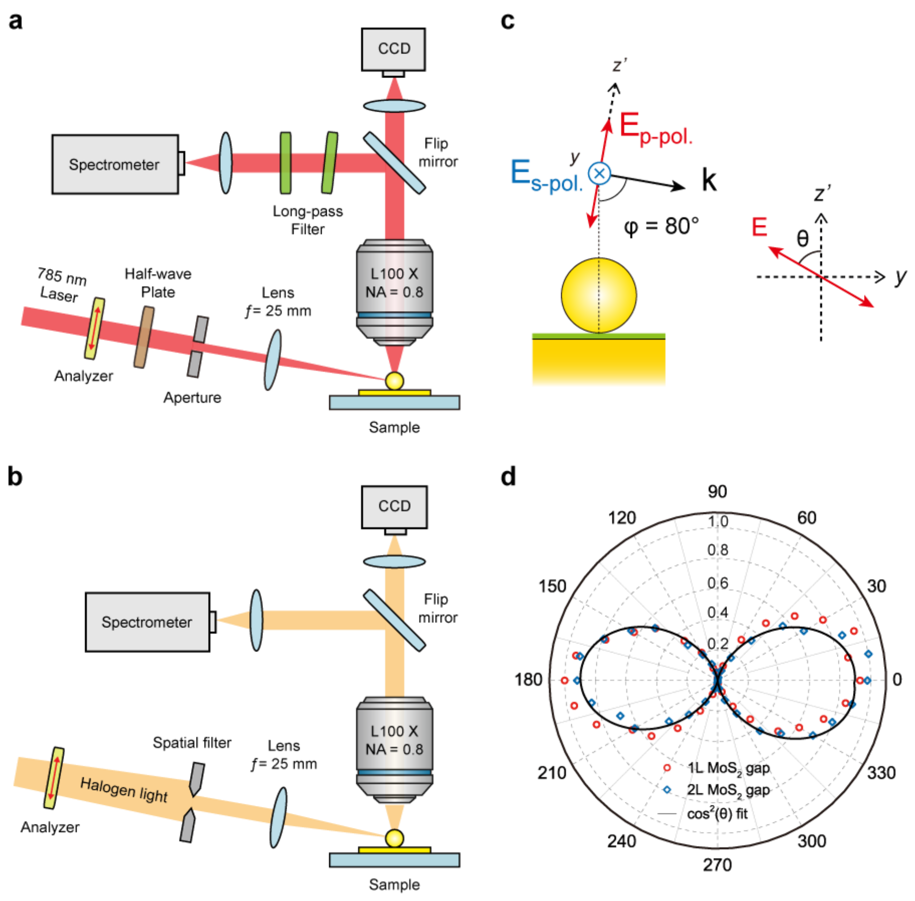

Supplementary Figure S4 Experimental setups and the incident polarization response of A_{1g}+A_{1g}' modes. (**a**, **b**) Light-path diagrams of SERS (**a**) and dark-field scattering (**b**) spectroscopy. (**c**) Oblique-excitation configuration for polarization dependent SERS and dark-field scattering measurements. (**d**) Relative intensity of A_{1g}+A_{1g}' modes as a function of the incident polarization angle θ, measured from a 1L (2L) MoS_2-NPOM with 32-nm-thick (38-nm-thick) Al_2O_3 coating. The curves were fitted by $\cos^2(\theta)$ function.

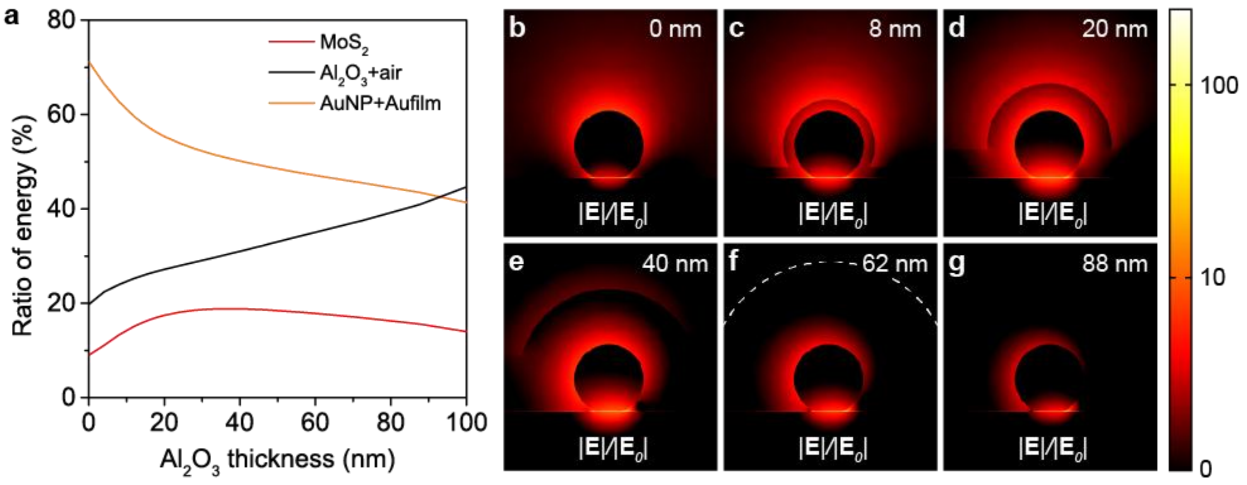

Supplementary Figure S5 Effect of Al_2O_3 coating on the plasmonic response of 1L MoS_2-NPOM. (**a**) The ratio of electromagnetic energy distributed within the MoS_2 layer, the Au structures and the dielectric space as functions of the Al_2O_3 thickness. (**b-g**) The near-field distributions of the 1L MoS_2-NPOM with Al_2O_3 coating thickness of 0 nm (**b**), 8 nm (**c**), 20 nm (**d**), 40 nm (**e**), 62 nm (**f**) and 88 nm (**g**). As the Al_2O_3 thickness increasing, the ratio of electromagnetic energy within the MoS_2 layer is firstly increasing and then gently decreasing, with the maximum of ~19% obtained at 36 nm. This feature matches well with the simulated Al_2O_3 thickness dependent SERS EFs shown in Figure S9, suggesting that the variation of the energy ratio within the MoS_2 is mainly caused by the plasmonic resonance excitation effect. The increase of the Al_2O_3 thickness also changes the dielectric screening effect, resulting in the electromagnetic energy transfer from the gold to the Al_2O_3 and air.

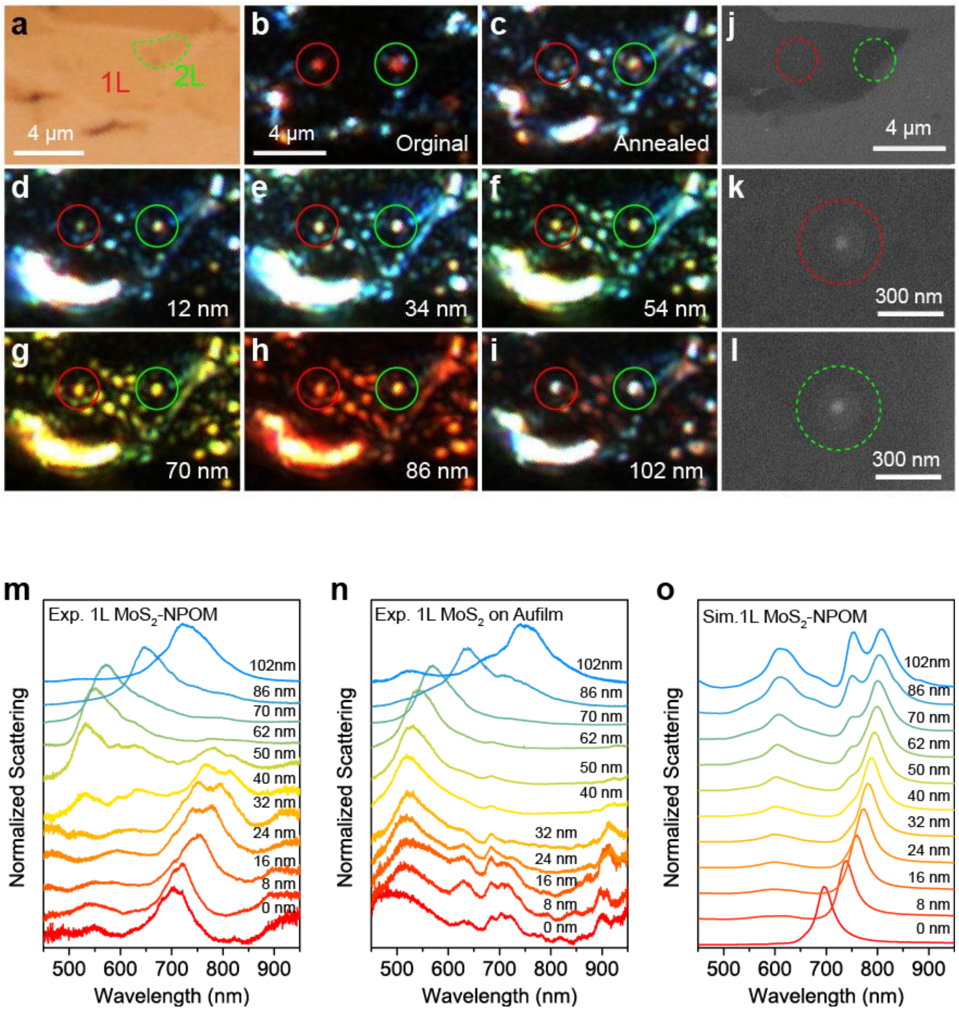

Supplementary Figure S6 SEM characterizations and dark-field scattering spectra of MoS_2-NPOMs. (**a**) Bright-field image of a MoS_2-NPOM without Al_2O_3 coating. (**b-i**) Dark-field scattering images of a 1L and a 2L MoS_2-NPOMs before annealing (**b**), after annealing with 0 nm (**c**), 12 nm (**d**), 34 nm (**e**), 54 nm (**f**), 70 nm (**g**), 86 nm (**h**) and 102 nm (**i**) thick Al_2O_3 surface coating. After annealing, bright spots appeared in the dark-field images, resulting from the scattered light of the MoS_2 bubbles (see Supplementary Figure S1 and S2). (**j-l**) Low- (**j**) and high-resolution (**k**, **l**) top view SEM images of the 1L (**k**) and 2L (**l**) MoS_2-NPOMs after 102-nm-thick Al_2O_3 coating. (**m**, **n**) Measured dark-field scattering spectra of a 1L MoS_2-NPOM (**m**, also shown in Figure 4a in the main text) and a 1L MoS_2 on ultrasmooth gold film (**o**) as the Al_2O_3 coating thickness varied from 0 nm to 102 nm. (**o**) The corresponding simulated scattering spectra of the 1L MoS_2-NPOM corresponding to (**m**). The peak around the 500 nm in (**o**) is identified as C' exciton, which always appears on MoS_2-over-mirror system[4]. As the Al_2O_3 thickness on the MoS_2-NPOMs exceeds ~50 nm, the intensity of the M and D

peaks both quickly reduce and finally become invisible. For further Al_2O_3 coating, a strong peak appears around the C' peak, whose redshift depends linearly on the Al_2O_3 coating thickness, with the intensity finally dominates the colors of the dark-field scattering images in (**b-i**). This feature cannot be observed in the simulations. It may come from the anti-reflective coating effect of the Al_2O_3 layer together with the scattered light from the wrinkles of the MoS_2. The small peak around 900 nm may come from the scattering light of the MoS_2 (second-order diffraction light has been removed).

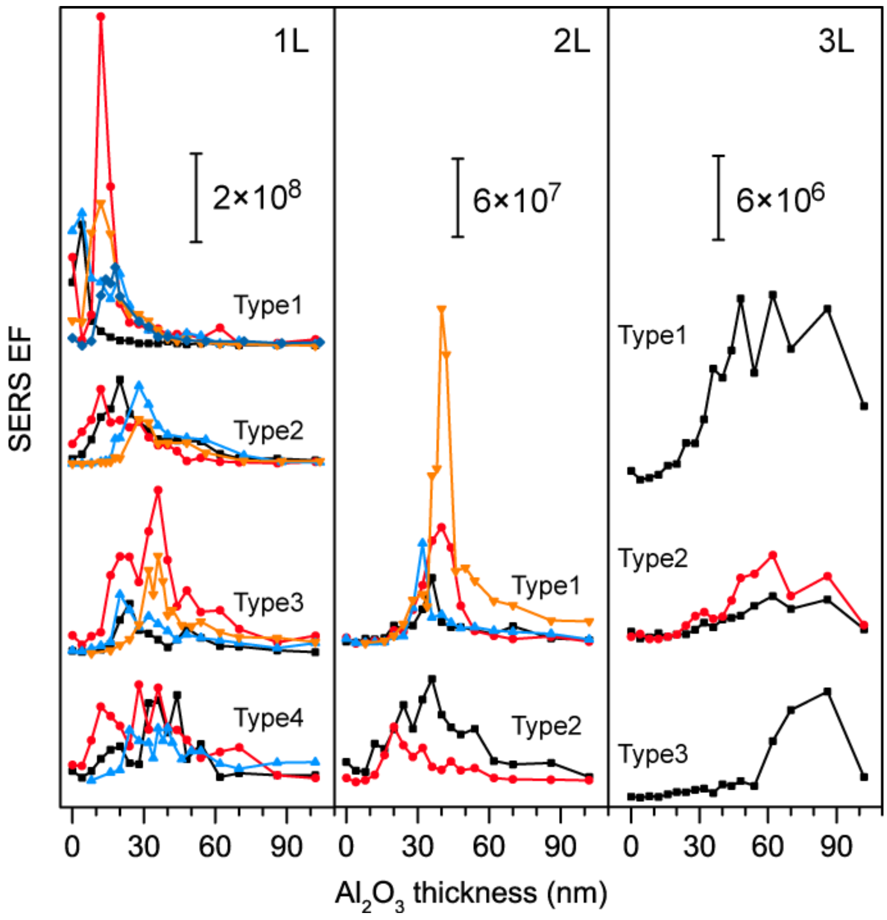

Supplementary Figure S7 Measured $\overline{\mathrm{EF}}_z$ of 1L, 2L and 3L MoS_2-NPOMs as a function of Al_2O_3 coating thickness. The results were measured from 17 individual 1L, 6 individual 2L and 4 individual 3L MoS_2-NPOMs. The maximum $\overline{\mathrm{EF}}_z$ in 1L, 2L and 3L MoS_2-NPOMs are 6.6×10^8, 2.2×10^8 and 1.1×10^7, respectively. All the profiles of the curves can be phenomenologically classified into in four typical types: 1) single peak, 2) single peak with a shoulder peak, 3) double peaks and 4) multiple peaks.

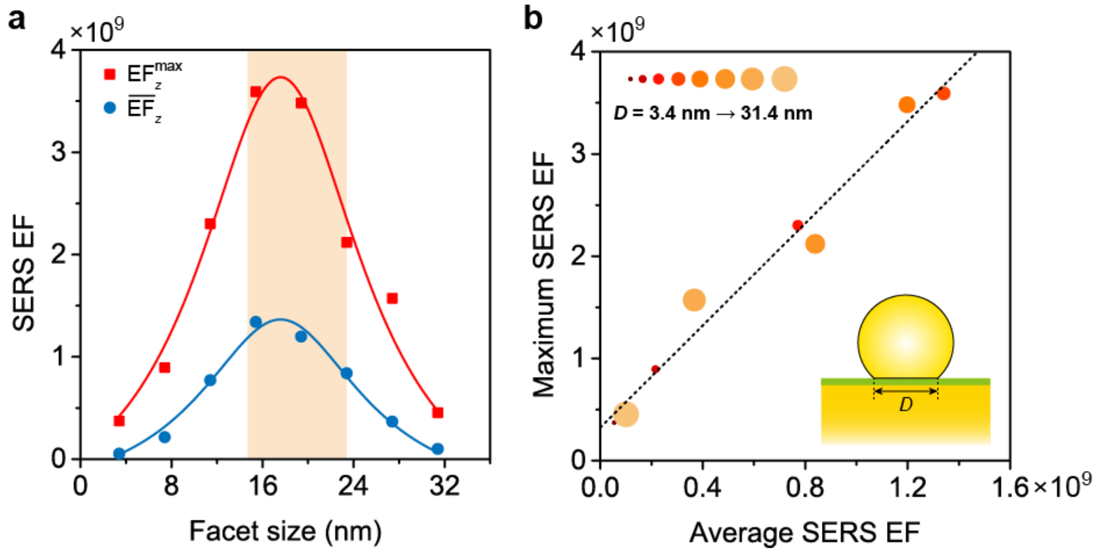

Supplementary Figure S8 Average and maximum SERS EFs in response to the AuNP's facet size. (**a**) $\overline{EF}_z$ and EF_z^{max} as a function of the diameter of the AuNP's bottom facet *D* in 1L MoS_2-NPOM system (fitted by Lorentz functions), where the EF_z^{max} is the maximum vertical SERS EF obtained from the point near the facet center (see Figure 3c in the main text). The shadow area shows the error range of the $\overline{EF}_z$ and EF_z^{max} as the diameter *D* is taken from the experimental range of 19.4±3.9 nm. It suggests that the error of the *D* could be considered as a main contribution for the error bars in experiments from Figure 5b and 6 in the main text. (**b**) Comparison between the $\overline{EF}_z$ and EF_z^{max} with different *D*. The linear fit gives a function of $EF_z^{max} = 3.24\times10^8 + 2.5\overline{EF}_z$. In our case of *D* = 19.4 nm, the measured (simulated) EF_z^{max} is 4.93×10^8 (3.48×10^9), thus the measured (simulated) g_z^{max} is 148 (243) based on the equation (1) in the main text.

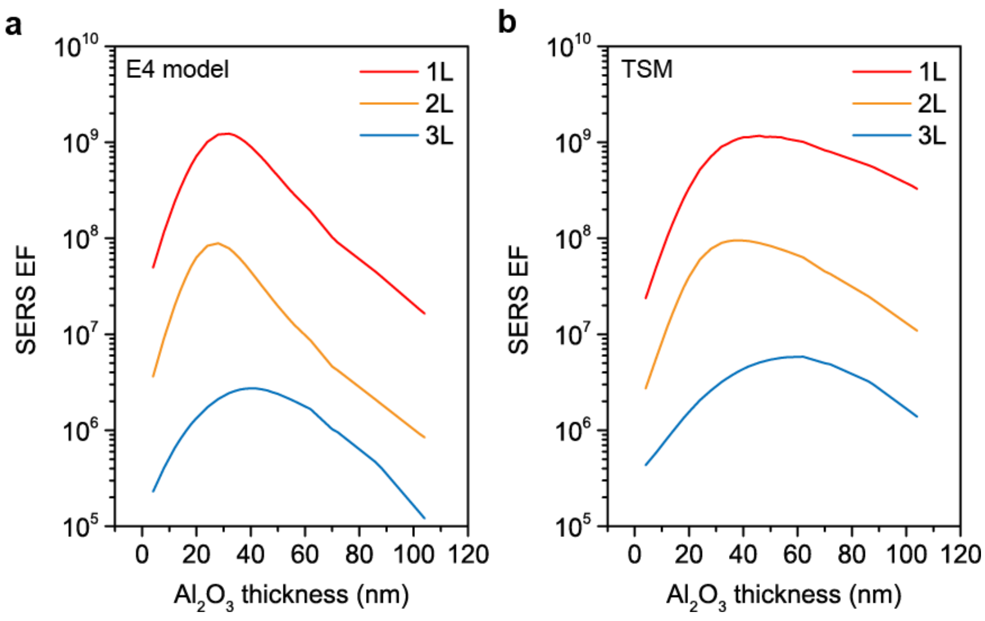

Supplementary Figure S9 Simulated SERS EFs of 1L, 2L and 3L MoS_2-NPOMs in response to Al_2O_3 coating thickness calculated by two electromagnetic models. (**a**) E4 model. (**b**) Two-study model (TSM). The detailed methods of these two models are shown in Materials and Methods, Supplementary Note S2. The maximum values of the three curves in (**a**) and (**b**) were used to plot the simulation results of Figure 6 in the main text.

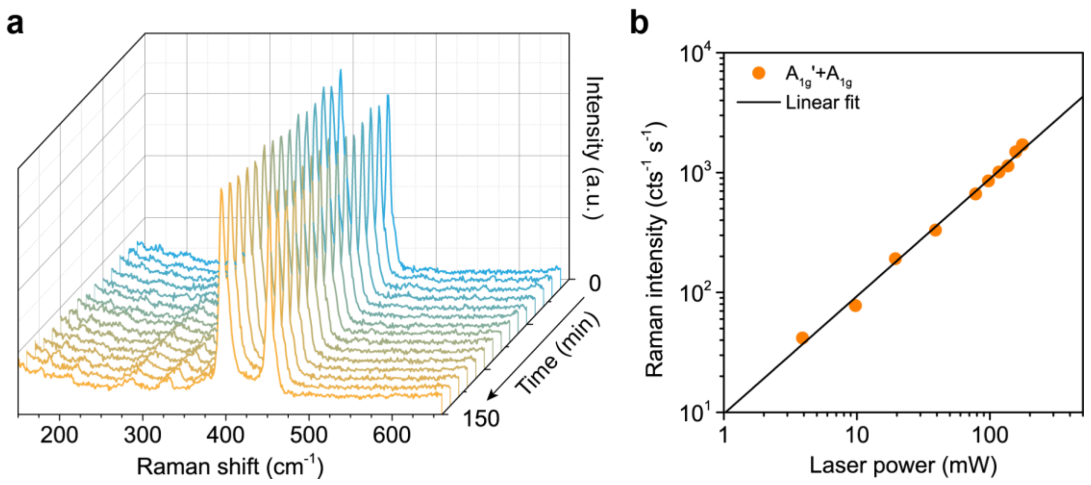

Supplementary Figure S10 Stability test and laser power dependence of the SERS intensity of MoS_2-NPOM system. (**a**) Time-dependent Raman profiles of a 1L MoS_2-NPOM system, showing 4.3% intensity error over 150 minutes. (**b**) SERS intensity of a 1L MoS_2-NPOM as a function of incident laser power. The result shows a linear relationship between the laser power and the SERS intensity.

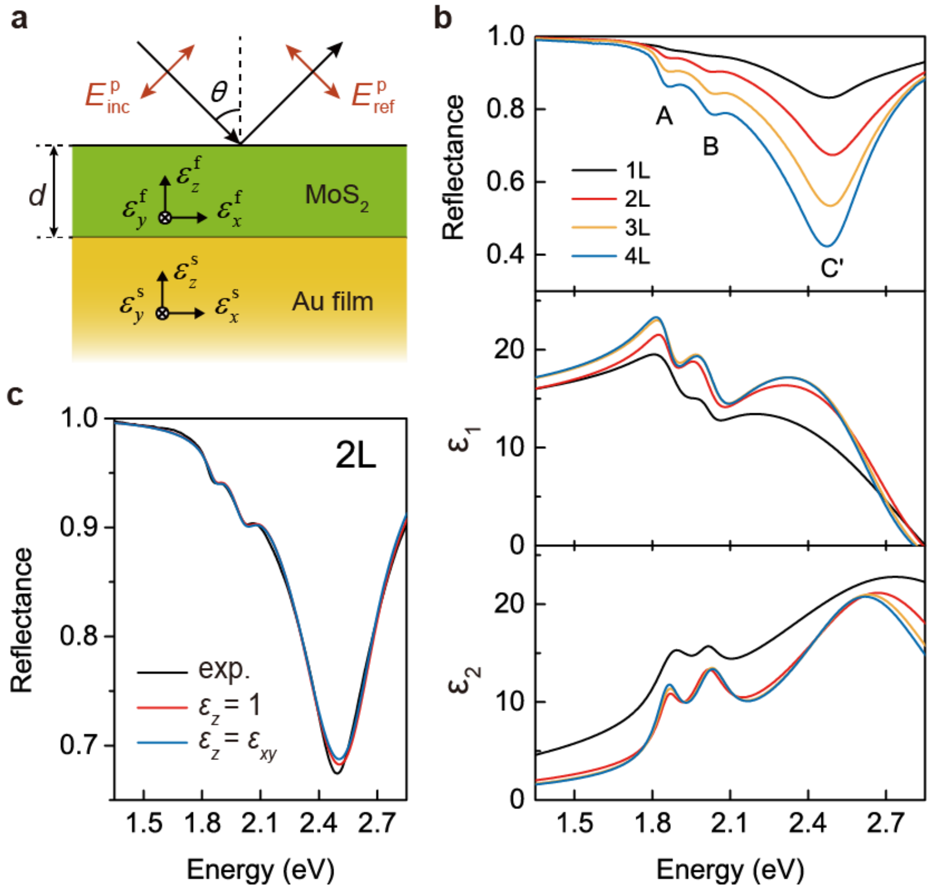

Supplementary Figure S11 Dielectric function measurements of single- and few-layer MoS_2 on gold film systems. (**a**) Schematic of reflectance configuration. (**b**) Reflectance spectra (top panel), real (middle panel) and imaginary (bottom panel) parts of the dielectric function of the 1L, 2L, 3L and 4L MoS_2 on ultrasmooth gold film. (**c**) The reflectance spectrum of the 2L MoS_2 and its corresponding fitting curves by setting different vertical components of the dielectric function.

Supplementary Notes

Supplementary Note S1. Analysis of vertical and horizontal SERS EFs:

For the Raman scattering process of MoS_2 on quartz, the incident field $\mathbf{E}_0(\omega)$ at frequency ω excites a Raman dipole $\mathbf{p}_0(\omega_\mathrm{R}) = \vec{\alpha}(\omega_\mathrm{R}, \omega)\mathbf{E}_0(\omega)$ at Raman frequency ω_R, where $\vec{\alpha}(\omega_\mathrm{R}, \omega)$ is the Raman polarizability tensor of the MoS_2. The scattered field $\mathbf{E}_\mathrm{Ref}(\omega_\mathrm{R})$ radiated by the Raman dipole $\mathbf{p}_0(\omega_\mathrm{R})$ can be described as:

$$\mathbf{E}_\mathrm{Ref}(\omega_\mathrm{R}) = \frac{\omega_\mathrm{R}^2}{\varepsilon_0 c^2}\int_\mathrm{qz} \overleftrightarrow{G}_0 \mathbf{p}(\omega_\mathrm{R})dV = \frac{\omega_\mathrm{R}^2}{\varepsilon_0 c^2}\int_\mathrm{qz} \overleftrightarrow{G}_0 \vec{\alpha}(\omega_\mathrm{R}, \omega)\mathbf{E}_0(\omega)dV \qquad \text{(S1)}$$

where ε_0 is the vacuum dielectric constant, c is the speed of light, $\overleftrightarrow{G}_0$ is the Green's function of the MoS_2 on quartz system. For the case of MoS_2 situated inside the NPOM gap region, a Raman dipole $\mathbf{p}(\omega_\mathrm{R})$ is induced by the local field $\mathbf{E}(\omega)$, forming the SERS field as the from:

$$\mathbf{E}_\mathrm{SERS}(\omega_\mathrm{R}) = \frac{\omega_\mathrm{R}^2}{\varepsilon_0 c^2}\int_\mathrm{NPOM} \overleftrightarrow{G} \mathbf{p}(\omega_\mathrm{R})dV = \frac{\omega_\mathrm{R}^2}{\varepsilon_0 c^2}\int_\mathrm{NPOM} \overleftrightarrow{G} \vec{\alpha}'(\omega_\mathrm{R}, \omega)\mathbf{E}(\omega)dV \qquad \text{(S2)}$$

where $\overleftrightarrow{G}$ is the Green's function of the MoS_2-NPOM system, $\vec{\alpha}'(\omega_\mathrm{R}, \omega)$ is the Raman polarizability tensor of the MoS_2 doped by Au. Here we assume that this doping effect doesn't bring significant influence on the SERS EFs, namely, $\vec{\alpha}'(\omega_\mathrm{R}, \omega) \approx \vec{\alpha}(\omega_\mathrm{R}, \omega)$. Then, the surface-averaged vertical and horizontal SERS EFs can be defined as:

$$\begin{aligned}\overline{\mathrm{EF}}_z &= \frac{I_\mathrm{SERS}^\mathrm{A} / S_\mathrm{SERS}^z}{I_\mathrm{Ref}^\mathrm{A} / S_\mathrm{Ref}} = \frac{\left|\mathbf{E}_\mathrm{SERS}^\mathrm{A}(\omega_\mathrm{R})\right|^2}{\left|\mathbf{E}_\mathrm{Ref}^\mathrm{A}(\omega_\mathrm{R})\right|^2}\cdot\frac{S_\mathrm{Ref}}{S_\mathrm{SERS}^z} \\ &= \frac{\left|\int_{V_z} \overleftrightarrow{G}\vec{\alpha}^\mathrm{A}(\omega_\mathrm{R}, \omega)\mathbf{E}(\omega)dV\right|^2}{\left|\int_{V_\mathrm{Ref}} \overleftrightarrow{G}_0\vec{\alpha}^\mathrm{A}(\omega_\mathrm{R}, \omega)\mathbf{E}_0(\omega)dV\right|^2}\cdot\frac{S_\mathrm{Ref}}{S_\mathrm{SERS}^z} = \frac{\left|\int_{V_z} \overleftrightarrow{G}\vec{\alpha}^\mathrm{A}(\omega_\mathrm{R}, \omega)\mathbf{E}(\omega)dV\right|^2}{\left|\int_{V_z} \overleftrightarrow{G}_0\vec{\alpha}^\mathrm{A}(\omega_\mathrm{R}, \omega)\mathbf{E}_0(\omega)dV\right|^2}\end{aligned} \qquad \text{(S3)}$$

$$\overline{\mathrm{EF}}_{xy}=\frac{I_{\mathrm{SERS}}^{\mathrm{E}}/S_{\mathrm{SERS}}^{xy}}{I_{\mathrm{Ref}}^{\mathrm{E}}/S_{\mathrm{Ref}}}=\frac{\left|\mathbf{E}_{\mathrm{SERS}}^{\mathrm{E}}(\omega_{\mathrm{R}})\right|^{2}}{\left|\mathbf{E}_{\mathrm{Ref}}^{\mathrm{E}}(\omega_{\mathrm{R}})\right|^{2}}\cdot\frac{S_{\mathrm{Ref}}}{S_{\mathrm{SERS}}^{xy}}$$

$$=\frac{\left|\int_{V_{xy}}\vec{G}\vec{\alpha}^{\mathrm{E}}(\omega_{\mathrm{R}},\omega)\mathbf{E}(\omega)dV\right|^{2}}{\left|\int_{V_{\mathrm{Ref}}}\vec{G}_{0}\vec{\alpha}^{\mathrm{E}}(\omega_{\mathrm{R}},\omega)\mathbf{E}_{0}(\omega)dV\right|^{2}}\cdot\frac{S_{\mathrm{Ref}}}{S_{\mathrm{SERS}}^{xy}}=\frac{\left|\int_{V_{xy}}\vec{G}\vec{\alpha}^{\mathrm{E}}(\omega_{\mathrm{R}},\omega)\mathbf{E}(\omega)dV\right|^{2}}{\left|\int_{V_{xy}}\vec{G}_{0}\vec{\alpha}^{\mathrm{E}}(\omega_{\mathrm{R}},\omega)\mathbf{E}_{0}(\omega)dV\right|^{2}} \tag{S4}$$

where $I_{\mathrm{SERS}}^{\mathrm{A}}$ ($\mathbf{E}_{\mathrm{SERS}}^{\mathrm{A}}(\omega_{\mathrm{R}})$) and $I_{\mathrm{SERS}}^{\mathrm{E}}$ ($\mathbf{E}_{\mathrm{SERS}}^{\mathrm{E}}(\omega_{\mathrm{R}})$) are the intensity (electric field) of the A_{1g}+A_{1g}’ and E_{2g}^{1}+E_{2g}^{1}’ modes of the MoS_2-NPOM system, $I_{\mathrm{Ref}}^{\mathrm{A}}$ ($\mathbf{E}_{\mathrm{Ref}}^{\mathrm{A}}(\omega_{\mathrm{R}})$) and $I_{\mathrm{Ref}}^{\mathrm{E}}$ ($\mathbf{E}_{\mathrm{Ref}}^{\mathrm{E}}(\omega_{\mathrm{R}})$) are the intensity (electric field) of the A_{1g} and E_{2g}^{1} modes obtained from the MoS_2 on quartz, $\vec{\alpha}^{\mathrm{A}}(\omega_{\mathrm{R}},\omega)$ and $\vec{\alpha}^{\mathrm{E}}(\omega_{\mathrm{R}},\omega)$ are the Raman polarizability tensors of the A_{1g} and E_{2g}^{1} modes, S_{SERS}^{z} (S_{SERS}^{xy}) and V_z (V_{xy}) are the effective area and the integral volume of the MoS_2 excited by the vertical (horizontal) local field, S_{Ref} (V_{Ref}) is the excitation area (integral volume) of the same layer MoS_2 on quartz. For our experiment, the vertical component is dominant both in the incident field $\mathbf{E}_0(\omega)$ (p-polarized) and the local field $\mathbf{E}(\omega)$. Therefore, in the SERS processes of the A_{1g}+A_{1g}’ modes we assume that the surface-averaged incoming (outgoing) vertical field enhancement factor $\overline{g_z^1}(\omega)$ ($\overline{g_z^2}(\omega_{\mathrm{R}})$) can be qualitatively extracted from the ratio of $\mathbf{E}(\omega)$ to $\mathbf{E}_0(\omega)$ ($\vec{G}$ to $\vec{G}_0$)[5]. As a result, the equation (S3) is reduced to:

$$\overline{\mathrm{EF}}_{z}\approx\left|\overline{g_z^1}(\omega)\right|^{2}\left|\overline{g_z^2}(\omega_{\mathrm{R}})\right|^{2} \tag{S5}$$

Due to the E_{2g}^{1} and E_{2g}^{1}’ modes can only be activated by the horizontal field, the corresponding in-plane field enhancement factor $\overline{g_{xy}^1}(\omega)$ and $\overline{g_{xy}^2}(\omega_{\mathrm{R}})$ can also be qualitatively extracted in a similar manner, then the equation (S4) is reduced to:

$$\overline{\mathrm{EF}}_{xy}\approx\left|\overline{g_{xy}^1}(\omega)\right|^{2}\left|\overline{g_{xy}^2}(\omega_{\mathrm{R}})\right|^{2} \tag{S6}$$

In comparison with the plasmonic peak-width of our MoS_2-NPOM system (~200 meV), the Raman outgoing wavelength of ~810 nm (the A_{1g}+A_{1g}’ and E_{2g}^{1}+E_{2g}^{1}’) is close to the excitation wavelength of

785 nm. Therefore, the $\overline{\mathrm{EF}}_z$ and $\overline{\mathrm{EF}}_{xy}$ can be further reduced to $\overline{\mathrm{EF}}_z \approx \left|\overline{g}_z(\omega)\right|^4$ and $\overline{\mathrm{EF}}_{xy} \approx \left|\overline{g}_{xy}(\omega)\right|^4$.

Supplementary Note S2. Simulations based on two-study model:

In the simulations based on the E4 model, the surface-averaged SERS EF was determined by fourth power of the local field enhancement, which was directly extracted from the near-field by $\iint_{S^z_{\mathrm{SERS}}} \left|\mathbf{E}/\mathbf{E}_0\right|^4 \mathrm{d}s \Big/ \iint_{S^z_{\mathrm{SERS}}} \mathrm{d}s$. This means that the E4 model ignores the Raman emission process described in Supplementary Note S1. Here we developed a new classical model called TSM to both simulate the laser excitation and Raman emission processes of the A_{1g} mode occurred in the MoS_2-NPOM and the MoS_2 on quartz systems. In TSM, we firstly calculated the local field distributions of the MoS_2-NPOM system, the 'first-study' that is the same as the E4 model. Then in the 'second-study', the emission field at Raman frequency excited by the local field was calculated by modeling the MoS_2 layer as an external polarization **P**. Finally, the SERS signal of the A_{1g} mode in the MoS_2-NPOM $I^{\mathrm{A}}_{\mathrm{SERS}}$ was computed by integrating all of the outgoing Raman scattering on the surface around the MoS_2 layer. For the MoS_2 on quartz system, the Raman intensity of the A_{1g} mode $I^{\mathrm{A}}_{\mathrm{Ref}}$ was obtained in a similar manner. Then the vertical SERS EF can be calculated by $(I^{\mathrm{A}}_{\mathrm{SERS}} \big/ S^{\mathrm{z}}_{\mathrm{SERS}}) / (I^{\mathrm{A}}_{\mathrm{Ref}} \big/ S_{\mathrm{Ref}})$, the same equation as used in experiments, where $S^{\mathrm{z}}_{\mathrm{SERS}}$ (S_{Ref}) is the effective excitation area of the MoS_2-NPOM (the same layer MoS_2 on quartz).

It should be noted that the spatial coherent effect of optical phonons, characterized by the correlation length, significantly impacts the optical emission processes in near-field regime, thereby cannot be neglected in our nanocavity system. In the TSM, this coherent effect was qualitatively taken into accounted by setting the MoS_2 layer as a finite-sized disk, whose diameter equals to its correlation length since the polarization current is coherent within the MoS_2 sheet of finite size. The correlation length of the A_{1g} modes in 1L, 2L and 3L MoS_2 were determined by matching the SERS EF results of the TSM to that of the E4 model, which were estimated as 24 nm, 28 nm and 34 nm, respectively.

These assumed correlation lengths are comparable with the measured value of ~30 nm for the optical phonons in graphene[6]. The SERS EF obtained by the TSM is sensitive to the correlation length, thus in turn the TSM could be used to estimate the unknown correlation length from different nanocrystals by matching the TSM to the E4 model and/or the experiment.

Specifically, besides the MoS_2 size mentioned above, the structural parameters and excitation configuration (the 'first-study') are the same as the E4 model. The emission field of the A_{1g} mode from the MoS_2-NPOMs was calculated by modeling the MoS_2 layer as an externally generated polarization $\mathbf{p}(\omega_R) = \tilde{\alpha}(\omega_R, \omega)\mathbf{E}(\omega)$ at ω_R, where $\mathbf{E}(\omega)$ is the local electric field from the 'first-study', ω is the excitation frequency (785 nm); ω_R is the Raman frequency of the A_{1g} of the 1L, 2L and 3L MoS_2, whose Raman shifts were all set to 403.9 cm^{-1} for simplicity. $\tilde{\alpha}(\omega_R, \omega)$ is the Raman polarizability tensor of the A_{1g} mode, whose components were taken to $\alpha_{xx}^{A} = \alpha_{yy}^{A}$ = -2.237873×10^{-10} F m^{-1} and α_{zz}^{A} = -3.756307×10^{-11} F m^{-1} according to the previously work[6]. The A_{1g} intensity of the MoS_2 on quartz was calculated in the same way. The Raman emission light was collected by a solid angle integrated over total space. The size of the 1L, 2L and 3L MoS_2 disks, namely the diameters of S_{Ref}, were taken to 24 nm, 28 nm and 34 nm, respectively. The diameters of S_{SERS}^{z} in the 1L, 2L and 3L MoS_2-NPOMs were equal to that of the vertical local field (22 nm), which are larger than their correlation length of MoS_2. Therefore, the MoS_2 layer in the MoS_2-NPOM can still be treated as infinite film, as the same in the E4 model.

Supplementary Note S3. Dielectric function measurements and analysis of single- and few-layer MoS_2 on gold film systems:

The dielectric function of the gold doped MoS_2 layer used in our NPOM system was obtained by experimental reflectance spectra using Kramers-Kronig constrained analysis, where the dielectric functions were extracted by fitting the spectral curves via the software package RefFIT[7]. The samples consist of 1L, 2L, 3L or 4L MoS_2 layer on 200-nm-thick ultrasmooth gold film after annealing

treatment (see Materials and Methods). For the reflectance spectral measurements, the sample was illuminated by p-polarized broadband light from a halogen lamp focused by a 10× objective (Supplementary Figure S11a). The size of the light spot was ~2 μm with incident angle θ = 45°. The reflected light was collected by a 50× objective and filtered by a p-polarized analyzer before directed to a spectrometer. The measured spectra were normalized by reference spectrum obtained from the reflected light of the bare gold film under the same configuration.

In this micro-reflection measurement configuration, the samples were viewed as a homogeneous thin layer over an anisotropic semi-infinite substrate, whose reflectance spectra for p-polarization excitation can be fitted by[7]:

$$R_p = |r_p|^2 = \left|\frac{E_{ref}^{p}}{E_{inc}^{p}}\right|^2 = \left|\frac{r_p^{0f} + t_p r_p^{fs}}{1 + t_p r_p^{0f} r_p^{fs}}\right|^2 \tag{S7}$$

$$r_p^{0f} = \frac{\sqrt{1-\frac{\sin^2\theta}{\varepsilon_z^{f}}} - \sqrt{\varepsilon_x^{f}}\cos\theta}{\sqrt{1-\frac{\sin^2\theta}{\varepsilon_z^{f}}} + \sqrt{\varepsilon_x^{f}}\cos\theta} \tag{S8}$$

$$r_p^{fs} = \frac{\sqrt{\varepsilon_x^{f}}\sqrt{1-\frac{\sin^2\theta}{\varepsilon_z^{s}}} - \sqrt{\varepsilon_x^{s}}\sqrt{1-\frac{\sin^2\theta}{\varepsilon_z^{f}}}}{\sqrt{\varepsilon_x^{f}}\sqrt{1-\frac{\sin^2\theta}{\varepsilon_z^{s}}} + \sqrt{\varepsilon_x^{s}}\sqrt{1-\frac{\sin^2\theta}{\varepsilon_z^{f}}}} \tag{S9}$$

$$t_p = \exp\left(4\pi i\omega d\sqrt{\varepsilon_x^{f}}\sqrt{1-\frac{\sin^2\theta}{\varepsilon_z^{f}}}\right) \tag{S10}$$

where ω is the light frequency and θ is the incident angle from the surface normal, set to 45°; d is the thickness of the 1L, 2L, 3L and 4L MoS_2 layers, taken to 0.615 nm, 1.23 nm, 1.845 nm and 2.46 nm, respectively[1], given by the integral multiple of interlayer spacing of the bulk MoS_2. $\varepsilon_x^{f}, \varepsilon_y^{f}, \varepsilon_z^{f}$ ($\varepsilon_x^{s}, \varepsilon_y^{s}, \varepsilon_z^{s}$) are the dielectric functions of the MoS_2 film (Au substrate) in three directions. They can be modeled as frequency dependent complex dielectric function $\varepsilon(\omega) = \varepsilon_1(\omega) + i\varepsilon_2(\omega)$ by the superposition of Lorentzian oscillators:

$$\varepsilon(\omega)=\varepsilon_{\infty}+\sum_{m}\frac{f_m\omega_m^2}{\omega_m^2-\omega^2-i\omega\gamma_m} \quad \text{(S11)}$$

where ε_{∞} is the high-frequency dielectric constant, ω_m, f_m and γ_m are the resonance frequency, the reduced oscillator strength and the linewidth of the *m*th Lorentz oscillator, respectively. The dielectric function of the gold film $\varepsilon_{\mathrm{Au}}(\omega)=\varepsilon_{\mathrm{x}}^{\mathrm{s}}=\varepsilon_{\mathrm{y}}^{\mathrm{s}}=\varepsilon_{\mathrm{z}}^{\mathrm{s}}$ was determined by fitting the experimental results from Johnson and Christy[8]. Therefore, the remaining variates in equation (S11) are the in-plane components $\varepsilon_{\mathrm{in}}(\omega)=\varepsilon_{\mathrm{x}}^{\mathrm{s}}=\varepsilon_{\mathrm{y}}^{\mathrm{s}}$ and out-of-plane component $\varepsilon_{\mathrm{out}}(\omega)=\varepsilon_{\mathrm{z}}^{\mathrm{s}}$ of the dielectric function of the MoS_2. The $\varepsilon_{\mathrm{in}}(\omega)$ of the 1L, 2L, 3L and 4L MoS_2 are plotted in Supplementary Figure S11b, showing that the A and B excitons of the gold doped MoS_2 are quenched in different levels depending on the number of layers of the MoS_2. The C' exciton originates from the mirror effect of the C exciton[4], where the latter can almost be ignored in the fitting as its intensity is significantly weaker than the former intensity. The $\varepsilon_{\mathrm{out}}(\omega)$ of the 1L, 2L and 3L MoS_2 were treated as constant 1, 2 and 2.4, determined by matching the simulated far-field spectra to the corresponding experimental results. As shown in Supplementary Figure S11c, the $\varepsilon_{\mathrm{out}}(\omega)$ was also tuned to match the reflectance spectra combined with fixed $\varepsilon_{\mathrm{in}}(\omega)$, whereas the resulting change of the spectrum profile is negligible even when the $\varepsilon_{\mathrm{out}}(\omega)$ was changed from $\varepsilon_{\mathrm{out}}(\omega)=1$ to $\varepsilon_{\mathrm{out}}(\omega)=\varepsilon_{\mathrm{in}}(\omega)$. This is because the thickness of single- and few-layer MoS_2 is too small to be compared with the incident wavelength.

References

1 Li YL, Chernikov A, Zhang X, Rigosi A, Hill MH *et al.* Measurement of the optical dielectric function of monolayer transition-metal dichalcogenides: MoS_2, $MoSe_2$, WS_2, and WSe_2. *Phys. Rev. B* 2014; **90**: 205422.

2 Sigle DO, Mertens J, Herrmann LO, Bowman RW, Ithurria S *et al*. Monitoring morphological changes in 2D monolayer semiconductors using atom-thick plasmonic nanocavities. *ACS Nano* 2015; **9**: 825-830.

3 Chen SY, Zheng CX, Fuhrer MS, Yan J. Helicity-resolved Raman scattering of MoS_2, $MoSe_2$, WS_2, and WSe_2 atomic layers. *Nano Lett.* 2015; **15**: 2526-2532.

4 Mertens J, Shi YM, Molina-Sánchez A, Wirtz L, Yang HY *et al.* Excitons in a mirror: formation of "optical bilayers" using MoS_2 monolayers on gold substrates. *Appl. Phys. Lett.* 2014; **104**: 191105.

5 Novotny L, Hecht B. *Principles of nano-optics*. Cambridge university press, 2012.

6 Beams R, Cançado LG, Oh SH, Jorio A, Novotny L. Spatial coherence in near-field Raman scattering. *Phys. Rev. Lett.* 2014; **113**: 186101.

7 Kuzmenko AB. *Guide to Reffit: software to fit optical spectra*. 2007; http://optics.unige.ch/alexey/reffit.html.

8 Johnson PB, Christy RW. Optical constants of the noble metals. *Phys. Rev. B* 1972; **6**: 4370-4379.
